# Association of Intravenous Thrombolysis with Delayed Reperfusion After Incomplete Mechanical Thrombectomy

**DOI:** 10.1007/s00062-022-01186-7

**Published:** 2022-07-14

**Authors:** Adnan Mujanovic, Christoph Kammer, Christoph C. Kurmann, Lorenz Grunder, Morin Beyeler, Matthias F. Lang, Eike I. Piechowiak, Thomas R. Meinel, Simon Jung, William Almiri, Sara Pilgram-Pastor, Angelika Hoffmann, David J. Seiffge, Mirjam R. Heldner, Tomas Dobrocky, Pasquale Mordasini, Marcel Arnold, Jan Gralla, Urs Fischer, Johannes Kaesmacher

**Affiliations:** 1 University Institute of Diagnostic and Interventional Neuroradiology, University Hospital Bern, Inselspital, University of Bern, Bern, Switzerland; 2 University Institute of Diagnostic, Interventional and Pediatric Radiology, University Hospital Bern, Inselspital, University of Bern, Bern, Switzerland; 3 Department of Neurology, University Hospital Bern, Inselspital, University of Bern, Bern, Switzerland; 4 Department of Neuroradiology, Heidelberg University Hospital, Heidelberg, Germany; 5 Department of Neurology, University Hospital Basel, Basel, Switzerland

**Keywords:** Ischemic stroke, Perfusion imaging, Outcome, Incomplete Reperfusion, tPA, Tissue-type plasminogen activator

## Abstract

**Purpose:**

Treatment of distal vessel occlusions causing incomplete reperfusion after mechanical thrombectomy (MT) is debated. We hypothesized that pretreatment with intravenous thrombolysis (IVT) may facilitate delayed reperfusion (DR) of residual vessel occlusions causing incomplete reperfusion after MT.

**Methods:**

Retrospective analysis of patients with incomplete reperfusion after MT, defined as extended thrombolysis in cerebral infarction (eTICI) 2a–2c, and available perfusion follow-up imaging at 24 ± 12 h after MT. DR was defined as absence of any perfusion deficit on time-sensitive perfusion maps, indicating the absence of any residual occlusion. The association of IVT with the occurrence of DR was evaluated using a logistic regression analysis adjusted for confounders. Sensitivity analyses based on IVT timing (time between IVT start and the occurrence incomplete reperfusion following MT) were performed.

**Results:**

In 368 included patients (median age 73.7 years, 51.1% female), DR occurred in 225 (61.1%). Atrial fibrillation, higher eTICI grade, better collateral status and longer intervention-to-follow-up time were all associated with DR. IVT did not show an association with the occurrence of DR (aOR 0.80, 95% CI 0.44–1.46, even in time-sensitive strata, aOR 2.28 [95% CI 0.65–9.23] and aOR 1.53 [95% CI 0.52–4.73] for IVT to incomplete reperfusion following MT timing <80 and <100 min, respectively).

**Conclusion:**

A DR occurred in 60% of patients with incomplete MT at ~24 h and did not seem to occur more often in patients receiving pretreatment IVT. Further research on potential associations of IVT and DR after MT is required.

**Supplementary Information:**

The online version of this article (10.1007/s00062-022-01186-7) contains supplementary material, which is available to authorized users.

## Introduction

The value of intravenous thrombolysis (IVT) in patients eligible for mechanical thrombectomy (MT), who have been directly referred to a comprehensive stroke center, remains debated [1, 2]. Most meta-analyses of observational studies have found an association between IVT and good functional outcomes [3–5]; however, recently published randomized-controlled trials could not corroborate this association, showing comparable outcomes in patients treated with direct MT, and those undergoing MT together with IVT [6–11]. Theoretical considerations have been made that a subset of patients may benefit from MT together with IVT, whereas others may benefit more from a direct MT approach [12].

Currently it is unclear if and how patients with incomplete reperfusion after MT should be treated further [13, 14]. Generally, data on incomplete reperfusion are heterogeneous and sparse [15–17]. Subanalysis of the Diffusion and Perfusion Imaging Evaluation for Understanding Stroke Evolution (DEFUSE) II trial showed good correlation between incomplete reperfusion at the end of MT and sustained incomplete reperfusion at 12‑h follow-up imaging [15], while a prospective single-center study showed that perfusion imaging could be used as a surrogate outcome marker after incomplete MT [16]. Other studies yielded findings with differing reperfusion rates after incomplete MT [17].

One potential advantage of IVT might be that it helps to recanalize residual occlusions after MT [1, 15–17]. We hypothesized that pretreatment with IVT and/or a running IVT infusion might facilitate delayed reperfusion (DR) of occlusions causing incomplete MT.

## Methods

A retrospective observational analysis of the University Hospital Bern prospective stroke registry was performed for all patients admitted with a diagnosis of acute ischemic stroke (AIS) between 2015 and 2020. This study was approved by the local ethics committee (reference ID 2020-01696) and conducted in accordance with the 1964 Helsinki Declaration and its subsequent amendments [18]. The data supporting the findings of this study are available from the corresponding author upon reasonable request and clearance by the ethics committee.

### Patient Selection

All patients undergoing MT for anterior circulation AIS between 1 February 2015 and December 8 2020 were evaluated, with the exception of patients who actively refused usage of their data for research purposes. After initial assessment by CT or MRI, only patients who were eligible for endovascular treatment according to our institution’s stroke guidelines were assessed. We restricted our analysis to internal carotid artery (ICA) and middle cerebral artery (MCA) occlusion patients with an incomplete reperfusion on final angiography series, defined as an expanded thrombolysis in cerebral infarction (eTICI) score of 2a–2c, and to those who had available follow-up perfusion imaging performed within 24 ± 12 h.

### Clinical Variables

Clinical variables and time intervals were extracted from the prospective Swiss Stroke Registry. In order to analyze the influence of IVT on DR at 24 ± 12 h after the intervention, comparison was made between patients receiving vs. not receiving IVT. All patients have received full IV tPA dose (0.9 mg/kg) without stopping the application before or during MT. Afterwards, additional sensitivity analysis dichotomized all patients into either of the two subgroups depending on the time between IVT start and the occurrence of incomplete MT, as seen on the final angiography imaging: (I) patients with short IVT intervals vs. (II) patients with long IVT intervals (Online Resources, Supplementary Figure 1). For example, when using an 80 min cut-off, based on alteplase terminal half-life [19], we compared all patients who had received IVT ≤ 80 min before the occurrence of incomplete MT, versus patients who had received IVT > 80 min before the occurrence of incomplete MT was noted on the final angiography run. The rationale behind this dichotomization was that patients with shorter time intervals between IVT start until the occurrence of incomplete MT were more likely to have therapeutic concentrations of circulating alteplase [19], while those with longer intervals between IVT start and the occurrence of incomplete MT were less likely.

Mortality and functional outcome were assessed with the modified Rankin Scale (mRS) score, where mRS 0–1 and mRS 0–2 were defined as excellent and good outcome, respectively. The mRS was assessed by an independent research nurse at 90-day follow-up visit, or via a structured telephone interview.

### Imaging

Neuroimaging was performed using standardized protocols upon admission, and subsequent follow-ups with either a CT (SOMATOM Definition Edge, Siemens, Erlangen, Germany) or 1.5/3T MRI (Avanto, Avanto fit, Verio, Aera, Skyra fit and Vida, Siemens). CT included non-contrast CT (slice thickness: 1 mm and/or 3 mm), CT angiography in early arterial (slice thickness: 0.6 mm), late venous (slice thickness: 1 mm) phase, and perfusion imaging. MRI sequences included fluid-attenuated inversion recovery (FLAIR), intracranial time-of-flight angiography (TOF), diffusion-weighted imaging (DWI), susceptibility-weighted imaging (SWI), maximum intensity projection (mIP), contrast-enhanced cervical and intracranial angiography, gradient-echo dynamic susceptibility contrast (DSC) perfusion, and optional postcontrast T1-weighted imaging.

The following MRI perfusion maps were generated using the Olea Sphere Software environment (Olea Sphere v2.3; Olea Medical, La Ciotat, France): relative cerebral blood volume (rBV), time to maximum (Tmax), mean transit time (MTT), relative cerebral blood flow (rBF), time to peak (TTP), and temporal maximum intensity projection (tMIP) maps. CT perfusion images were processed by the postprocessing software syngo.via (Siemens). According to our institution’s internal protocols all patients undergo MRI including follow-up perfusion imaging as per clinical routine, and CT perfusion was only acquired if clinically indicated (e.g. non-hemorrhagic neurological worsening, lack of clinical improvement). There was no study-specific imaging and all analyzed data was acquired in clinical routine. DR was evaluated on the first follow-up imaging, which included perfusion imaging, using TTP and Tmax maps due to their high sensitivity rates [20–22].

Sites of arterial occlusions were categorized into one of the following categories, based on the initial imaging: intracranial carotid artery (ICA), proximal segment of the middle cerebral artery (M1), insular segment of the middle cerebral artery (M2), and opercular segment of the middle cerebral artery (M3). Reperfusion success was graded on the final angiogram series using the eTICI scale [23], with 2a, 2b50, 2b67, 2c and 3 referring to reperfusion of 1–49%, 50–66%, 67–89%, 90–99% and 100% of the affected territory, respectively. An eTICI ≥ 2b50 was categorized as successful reperfusion [23]. Reperfusion success was graded by four neuroradiologists who were not involved in interventions which were evaluated and were blinded to all clinical data. The eTICI score grading was performed according to the latest modification and detailed instructions outlined in the recent consensus statement [24]. Collateral status was graded with the American Society of Interventional and Therapeutic Neuroradiology/Society of Interventional Radiology (ASITN/SIR) scale on preinterventional angiography imaging [24].

### Delayed Reperfusion and Persistent Perfusion Deficit

**Fig. 1:**
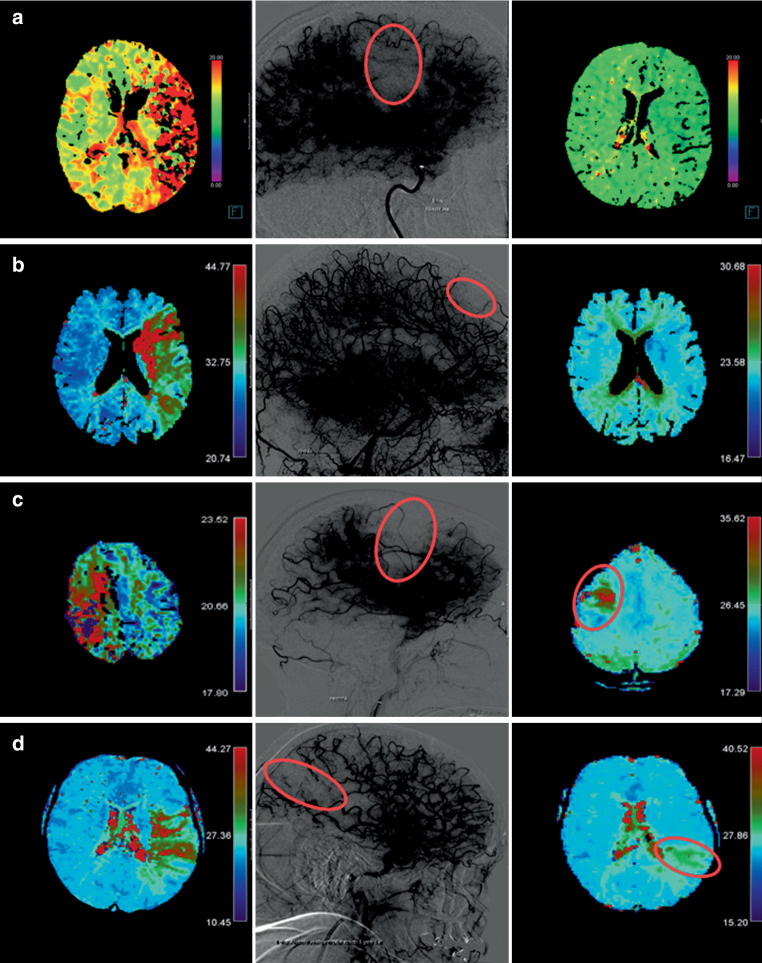
Delayed reperfusion (DR) and persistent perfusion deficit (PPD) on perfusion follow-up imaging. Final angiography runs (*middle panel*) are displayed with high contrast in order to emphasize the capillary phase deficits. TTP and Tmax perfusion imaging maps were evaluated on admission (*left panel*) and follow up (*right panel*) examinations due to their high sensitivity rates (Olea Sphere v2.3; Olea Medical & syngo.via, Siemens). **a** Patient with a left side M1 occlusion with DR on CT follow-up after extended thrombolysis in cerebral infarction (eTICI) 2b50 reperfusion. **b** Patient with a left side ICA occlusion with DR on MRI follow-up after eTICI 2c reperfusion. **c** Patient with a right side M2 occlusion with PPD on MRI follow-up after eTICI 2b50 reperfusion. **d** Patient with a left side M1 occlusion with PPD on MRI follow-up after eTICI 2b50 reperfusion

DR was the primary outcome of this study. It was defined as the absence of any focal perfusion deficit on time-sensitive maps of contrast-enhanced follow-up perfusion imaging (Fig. 1a,b). Conversely, persistent perfusion deficit (PPD) was defined as a persisting perfusion imaging deficit clearly visible on follow-up perfusion imaging (focally increased TTP and Tmax values on the ipsilateral, compared to the contralateral side), which corresponded to the location of the antegrade capillary phase deficit on the final angiography image of the incompletely perfused area after MT (Fig. 1c,d). Great care was taken to exclude false positive ratings of PPD caused by susceptibility artifacts/hemorrhagic transformations (Online Resources, Supplementary Figure 2). For this purpose, other sequences including SWI were assessed. Rating perfusion outcome as a dichotomized output was used with the aim of showing reliability of perfusion imaging outcome and its potential usefulness in future studies. Correspondingly, a qualitative approach offers simplified understanding of perfusion outcome for the readers and its practical use in clinical routine. Perfusion outcome was evaluated with centrally adjudicated charter distributed among the raters (Online Resources, Supplementary Methods 1). All qualitative ratings were performed by four neuroradiologists blinded to clinical data, with experience of > 20, > 15, > 5 and > 1 years. Interrater agreement was evaluated for a random sample of 50 patients.

### Statistical Analysis

Variables between the groups were compared using Mann-Whitney U and Fisher exact tests for continuous and categorical variables, respectively. Results are reported as median (interquartile range [IQR]) and *n* (%), unless specified otherwise. Multivariate logistic regression was carried out with the occurrence of DR as dependent variable. Results of the logistic regression analysis are displayed as adjusted odds ratios (aOR) and the corresponding 95% confidence intervals (CI). Regression was adjusted for baseline confounders and potentially pathophysiological covariates which could influence the occurrence of DR: age (continuous variable), sex (binary variable), atrial fibrillation (AF, binary variable), prestroke anticoagulant usage (binary variable), prestroke antiplatelet usage (binary variable), National Institutes of Health Stroke Scale (NIHSS) on admission (ordinal variable, aOR referring to one point increase), onset-to-door time (continuous variable, aOR referring to 1h delay), intervention-to-follow-up time (continuous variable, aOR referring to 1h delay), IVT usage (binary variable), collateral status (ordinal variable with a step-wise increase), maneuver count (ordinal variable with a step-wise increase) and eTICI score (categorical variable, allowing for changing aOR in each eTICI stratum compared to the baseline level = eTICI 2a). In order to exclude collinearity between independent variables and to check their strength of correlation, we have calculated the variance inflation factor (VIF), with VIF = 1 indicating no correlation, and VIF > 5 representing critical levels of multicollinearity. All variables included in our regression models had hardly detectable collinearity (VIF < 1.5). For illustration purposes, aORs of the independent variables were plotted as forest plots, and predicted probabilities were plotted with varying intervention-to-follow-up times using the logistic regression model, while holding all other variables constant (i.e. continuous variables were set to their mean and factor variables to their base levels).

In order to fully assess outcome effects several subanalyses were performed. Patients with eTICI 2c have relatively better clinical outcome compared to eTICI 2a–2b patients [25, 26]. In order to evaluate this in the context of perfusion imaging outcome, additional subanalysis excluded eTICI 2c patients and was restricted to eTICI 2a–2b67 patients only. Patients with reocclusions were excluded from the main analyses, as per our definition of PPD they do not present perfusion deficits limited to the area of small persistent occlusions (Supplementary Methods 1). For sensitivity purposes, a subanalysis was run with reocclusion patients being classified as patients with PPD. In order to analyze the influence of IVT timing and its possible association with DR, sensitivity models were performed for dichotomized IVT strata sets (see Clinical Variables for definition and rationale). Analyses of these different regression models are presented as a forest plot.

mRS scores at 90 days with strata of perfusion imaging outcomes (DR vs. PPD) are graphically displayed using bar plots. All outcome analyses have been adjusted for the following confounders: age, sex, NIHSS on admission, prestroke mRS, onset-to-door time and final eTICI reperfusion score.

All statistical analyses were conducted using R v4.0.0. [27] For a complete list of used statistical packages, refer to the supplement file (Online Resources, Supplementary Table 1).

## Results

### Study Population and Inclusion Bias

A total of 368 patients constituted the final study population (Online Resources, Supplementary Figure 3). Median age of the cohort was 73.7 years (IQR 61.4–81 years) and 188 (51.1%) were female. Patients presented with severe neurological symptoms (median NIHSS 14, IQR 8–19), with a median time delay of 3 h 12 min after symptom-onset (IQR 1 h 42 min–7 h 18 min).

An exploratory analysis for inclusion bias was performed comparing cohorts with follow-up perfusion imaging to those without. Undergoing a follow-up imaging with CT or MR perfusion was associated with a better baseline characteristics (Online Resources, Supplementary Table 2).

### Delayed Reperfusion and Associated Factors

Interrater agreement for the rating of DR was excellent (Krippendorf’s alpha 0.82, 95% CI 0.80–0.84). In the complete cohort, DR occurred in 225 patients (61.1%), and in 82 (60.3%) of patients who received IVT. Median intervention-to-follow-up time was 21 h 6 min (IQR 16 h 54 min–24 h 18 min), with 75.3% of patients undergoing MRI and 24.7% CT perfusion at follow-up.

When comparing patients with DR versus those with PPD, we found that DR was more likely to occur in patients with AF (DR: 38.7%; PPD: 27.3%; *p* = 0.033), longer intervention-to-follow-up time (DR: 21 h 54 min [IQR 18 h 30 min–25 h 18 min]; PPD: 19 h 30 min [IQR 14 h 48 min–22 h 54 min]; *p* < 0.001) and lower number of device passes (DR: 1 [IQR 1–2]; PPD: 2 [IQR 1–3]; *p* < 0.001). In addition, patients with better reperfusion scores were more likely to achieve DR than patients with poor incomplete reperfusion scores (e.g. rate of DR eTICI 2c vs. rate of DR eTICI 2a, *p* < 0.001). This effect could be observed even when performing a step-based comparison between each eTICI score denoting incomplete reperfusion: eTICI 2a: 21.1% vs. eTICI 2b50: 20% vs. eTICI 2b67: 59.1% vs. eTICI 2c: 82.8%; *p* = 0.001. A complete overview of baseline, intervention and outcome parameters is shown in Table 1.

**Table 1:** Patients stratified by perfusion imaging outcome

	Overall	Delayed reperfusion	Persistent perfusion deficit	*p*
*n*	368	225	143	–
**Baseline**				
*Age (years)*	73.7 (61.4, 81)	73.7 (63.6, 80.3)	73.6 (60.6, 81.8)	0.745
*Sex (female)*	188 (51.1)	112 (49.8)	76 (53.1)	0.601
**Medical History**				
*Atrial fibrillation*	126 (34.2)	87 (38.7)	39 (27.3)	0.033
*Coronary heart disease*	63 (17.1)	45 (20.0)	18 (12.6)	0.089
*Diabetes*	55 (14.9)	34 (15.1)	21 (14.7)	1
*Hyperlipidemia*	236 (64.1)	145 (64.4)	91 (63.6)	0.963
*Hypertension*	250 (67.9)	161 (71.6)	89 (62.2)	0.080
*Current smoking status*	73 (19.8)	42 (18.7)	31 (21.7)	0.567
*Previous stroke*	42 (11.4)	22 (9.8)	20 (14.0)	0.285
*Previous TIA*	19 (5.2)	11 (4.9)	8 (5.6)	0.955
*Glucose on admission (mmol/L)*	6.5 (5.8, 8)	6.4 (5.8, 8.2)	6.7 (5.7, 7.8)	0.736
*Anticoagulants prestroke*	55 (14.9)	31 (13.8)	24 (16.8)	0.523
*Antiplatelets prestroke*	106 (28.8)	63 (28.0)	43 (30.1)	0.407
*mRS 0–1 prestroke*	299 (81.2)	190 (84.4)	109 (76.2)	0.067
*NIHSS on admission*	14 (8, 19)	14 (8, 18)	13 (8, 19)	0.700
**Baseline imaging findings**				
*Occlusion site*	–	–	–	0.585
ICA	75 (20.4)	47 (20.9)	28 (19.6)	–
M1	195 (52.5)	120 (53.3)	72 (50.3)	–
M2	96 (26.1)	54 (24.0)	42 (29.4)	–
M3	5 (1.4)	4 (1.8)	1 (0.7)	–
*ASITN/SIR collateral score*	1 (1, 2)	1 (1, 3)	1 (1, 2)	0.001
**Intervention**				
*Onset-to-door (h)*	3.2 (1.7, 7.3)	3.3 (1.8, 7.2)	3.0 (1.7, 7.4)	0.891
*Intervention-to-follow-up (h)*	21.1 (16.9, 24.3)	21.9 (18.5, 25.3)	19.5 (14.8, 22.9)	< 0.001
*Intravenous thrombolysis*	136 (37.0)	82 (36.4)	54 (37.8)	0.885
*Number of device passes*	1 (1, 3)	1 (1, 2)	2 (1, 3)	< 0.001
**Outcome**				
*Imaging modality on follow-up*	–	–	–	< 0.001
*CT*	91 (24.7)	42 (18.7)	49 (34.3)	–
*MRI*	277 (75.3)	186 (81.3)	94 (65.7)	–
*mRS at 90 days*	2 (1, 5)	2 (1, 4)	3 (2, 5)	< 0.001
*Mortality at 90 days*	55 (14.9)	27 (12.0)	28 (19.6)	0.052

Categorical data are expressed as real numbers (*n*) and percentages (%). Continuous data are presented as median (*n*) and interquartile range (IQR). *P*-values represent group-wise comparisons of patients with delayed reperfusion and persistent perfusion deficit with the selection of tests outlined in the Methods section. Missing values were present in the case of following variables: glucose on admission *n* = 1 (0.3%), onset-to-door time (h) *n* = 10 (2.7%), number of device passes *n* = 6 (1.6%) and mortality at 90 days *n* = 31 (8.4%)

*TIA* transitory ischemic attack, *mRS* modified Rankin Scale, *NIHSS* National Institutes of Health Stroke Scale, *ICA* intracranial carotid artery occlusion, *eTICI* extended Thrombolysis in Cerebral Infarction, *M1* Proximal Occlusion of the Middle Cerebral Artery, *M2* Insular Segment Occlusion of the Middle Cerebral Artery, *M3* Opercular Segment Occlusion of the Middle Cerebral Artery, *ASITN/SIR *American Society of Interventional and Therapeutic Neuroradiology/Society of Interventional Radiology

**Fig. 2:**
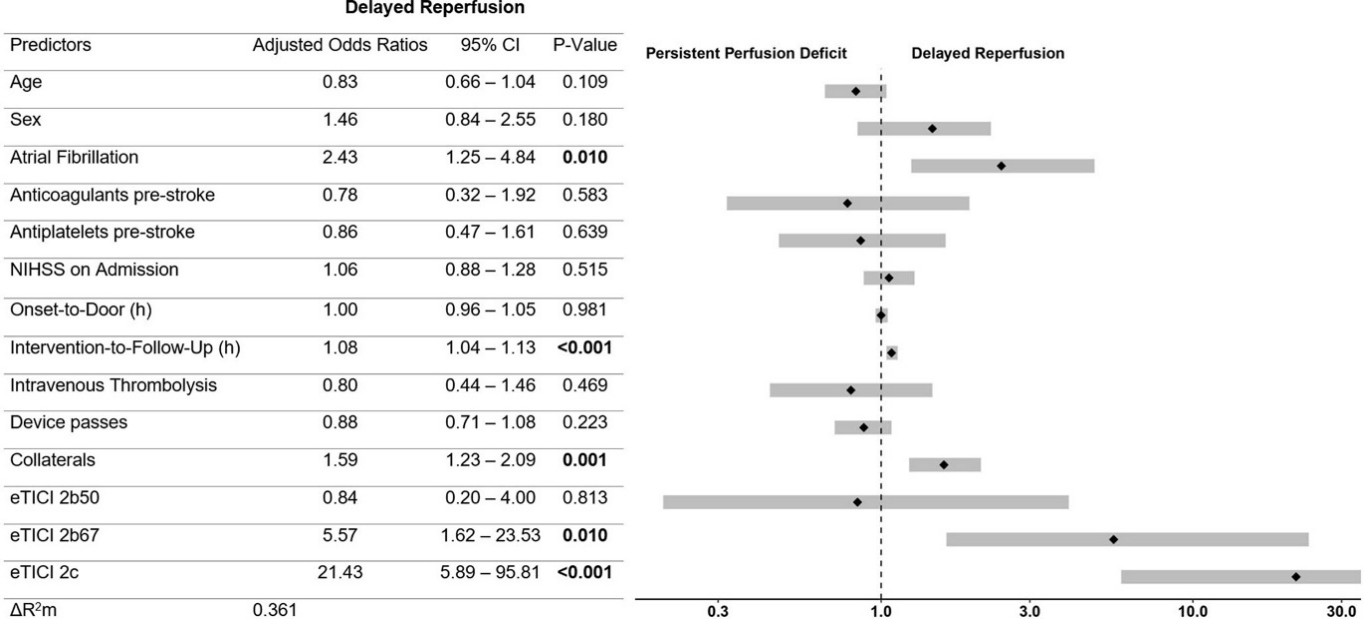
Logistic regression model with delayed reperfusion as a dependent variable. Adjusted odds ratios (aOR) for variable “Age” refer to 10-year increase and aOR for variable “NIHSS on Admission” to a 5-point increase. After adjusting for confounders, fitted multivariable logistic regression model for perfusion imaging outcome reveals the following significant associations: atrial fibrillation, eTICI score, collateral status, and intervention-to-follow-up time. *NIHSS* National Institutes of Health Stroke Scale, *eTICI* extended thrombolysis in cerebral infarction

After adjusting for confounders, logistic regression analysis revealed the following significant associations with DR (Fig. 2): AF (aOR 2.43 [95% CI 1.25–4.84]), higher eTICI score (e.g. eTICI 2b67 aOR 5.57 [95% CI 1.62–23.53], comparator eTICI 2a), better collateral status (aOR 1.59 [95% CI 1.23–2.09] per grade increase), and longer intervention-to-follow-up time. The magnitude of the latter was an 8% relative increase of the occurrence of DR per additional hour delay of follow-up imaging (aOR 1.08 [95% CI 1.04–1.13], Online Resources, Supplementary Figure 4). On a dichotomized analysis (patients with and without IVT), pretreatment with IVT was not associated with DR (aOR 0.80 [95% CI 0.44–1.46]). Collateral status showed a strong association with achieving DR in both patients who have received IVT and in those who did not (aOR 1.78 [95% CI 1.08–3.16], aOR 1.50 [95% CI 1.09–2.08], respectively; Supplementary Table 3). Sensitivity analyses which classified reocclusions as PPD and/or were confined to only 2a–2b67 cases, yielded comparable results (Online Resources, Supplementary Table 4).

**Fig. 3:**
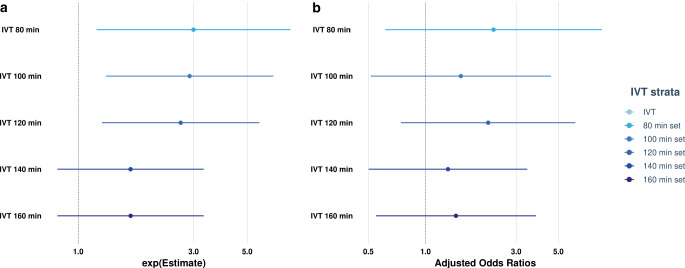
Sensitivity regression model of different intravenous thrombolysis sets. Log odds are calculated by applying regression models on perfusion imaging outcome after the intervention, for both unadjusted (**a**) and adjusted (**b**) analyses with additional covariates (see Methodology). There are comparable results of IVT having an influence on follow-up imaging perfusion outcome in the range of shorter time windows. This is significant in the 80 and 100 min unadjusted strata set (OR 2.99 [95% CI 1.24–8.07], and OR 2.88 [95% 1.33–6.63], respectively). Adjusted analyses failed to show this association for 80 and 100 min strata sets (aOR 2.28 [95% CI 0.65–9.23] and aOR 1.53 [95% CI 0.52–4.73], respectively). *IVT* intravenous thrombolysis

Acknowledging different timeframes between IVT administration and the occurrence of incomplete reperfusion following MT, several subanalyses were performed. On an unadjusted analysis, patients receiving IVT in shorter time windows before the occurrence of incomplete MT showed an association with achieving DR (OR 2.99 [95% CI 1.24–8.07], and OR 2.88 [95% 1.33–6.63], for IVT start time ≤80 and ≤100 min before the occurrence of incomplete MT, respectively, Fig. 3a). After including all the covariates outlined in the Methodology section (see Statistical Analyses), adjusted model failed to show this association for DR in the ≤80 and ≤100 min strata sets (aOR 2.28 [95% CI 0.65–9.23] and aOR 1.53 [95% CI 0.52–4.73] respectively, Fig. 3b and Online Resources, Supplementary Table 5).

### Outcome

**Fig. 4:**
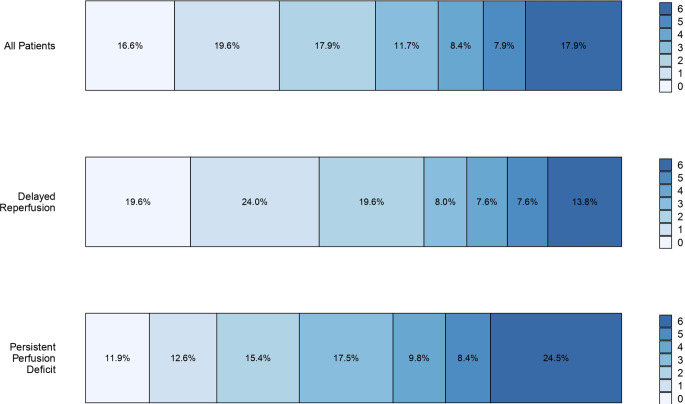
Functional outcomes stratified according to the perfusion imaging outcome. Excellent and good outcome were significantly more frequent in the delayed reperfusion group, as opposed to those who had persistent perfusion deficit (mRS 0–1 at 90 days: 43.6% vs. 24.5% *p* < 0.001; mRS 0–2 at 90 days: 63.2% vs. 39.9%, *p* < 0.001, respectively)

Functional outcomes differed significantly between patients with DR and PPD, with more patients achieving excellent (mRS 0–1 at 90 days: 43.6% vs. 24.5%, *p* < 0.001) and good outcome (mRS 0–2 at 90 days: 63.2% vs. 39.9%, *p* < 0.001, Fig. 4) in the DR group. The 3‑month mortality was 14.9% (DR: 12% vs. PPD: 19.6%). All of these associations remained after adjusting for factors outlined in the Methodology (aOR 1.80 [95% CI 1.01–3.37], aOR 2.50 [95% CI 1.35–4.71] and 0.70 [95% CI 0.31–1.60], for mRS 0–1, mRS 0–2, and mortality, respectively, Supplementary Table 6).

## Discussion

This study has the following main findings: (1) DR occurs in approximately 6 out of 10 patients after incomplete MT at 24 h, implying that more than 1/3 of patients have a PPD related to the residual occlusion after incomplete MT. (2) IVT does not show an association with the occurrence of DR. (3) DR is time-dependent, with reperfusions more frequently observed at longer intervention-to-follow-up times, and is strongly associated with favorable clinical outcomes. (4) Factors associated with DR were AF, and more prominently, better reperfusion scores across the eTICI spectrum.

### Perfusion Imaging after MT

Data from the DEFUSE II trial suggest that there is a good correlation between post-MT angiographic reperfusion and reperfusion at 12 h. [15] In particular, patients with TICI 2b–3 reperfusion (defined as ≥ 50% of the vascular distribution) had a more favorable clinical outcome, than TICI 0–2a patients (defined as < 50% of the vascular distribution), concluding that continued improvement after the intervention is more likely than a decline [15]; however, considerable heterogeneity of the reperfused penumbra area has been observed in TICI 2b patients (median percental reperfusion status: 90% IQR [15–100]).[15] In this study there were no data on correlations between the perfusion deficit observed on final angiography runs and follow-up imaging [15].

Rubiera et al. analyzed 151 patients who had received immediate CT perfusion 30 min after MT [16]. Corroborating the data from the DEFUSE II trial, they have also observed large heterogeneity regarding the volume of residual perfusion deficit after TICI 2b reperfusion (14 cubic centimeters (cc) [IQR 0 cc–38 cc]).[16] In addition, they provided strong evidence that patients with less perfusion deficit on immediate post-intervention CT perfusion had better outcome, and this was tangible when considering a specific TICI grade only (e.g. only patients with TICI 2b) [16]. Correspondingly, Brugnara et al. showed considerable heterogeneity within the volume change of the hypoperfused territory in TICI 2b reperfusions [17].

Reperfusion is a well known time-dependent phenomenon [28]. Our study shows that the heterogeneity of follow-up perfusion imaging deficit in TICI 2b patients may be partially explained by time-sensitive DR observed on angiography images. The more time has passed since the intervention, the higher the odds of DR being observed on the follow-up imaging. Presented inclusion bias could mainly be attributed to the postprocedural patient status, where patients who are doing poorly, are also usually too unfit to undergo perfusion imaging. Confirming the findings by Rubeira et al. [16] we have also found evidence of strong clinical effect of DR, showing that patients with DR had higher rates of functional outcome and lower long-term mortality. Considering strong clinical association and direct evaluation of the desired therapeutic effect, follow-up perfusion imaging may constitute an ideal surrogate parameter to evaluate the efficacy of additional rescue strategies after incomplete MT (e.g. administration of intra-arterial/intravenous thrombolytics) [14, 29].

While we cannot exclude that microcirculatory, rather than true persistent vessel occlusions, have caused PPD observed in our analysis, clinical data supporting true microcirculatory obstruction/reflow phenomena in patients are rather heterogeneous [15–17, 30, 31]. Heterogeneity in these observations may be explained by how persistent deficit and potential microcirculatory obstructions were defined and measured [15–17, 30, 31].

AF is associated with reduced spontaneous recanalization rates of the occluded large or middle cerebral arteries [32]. In our study we have observed an inverse relationship where AF was associated with improved rates of DR. It could be hypothesized that AF might be a proxy for another factor which impacts perfusion outcome, even though confounding was minimized by the inclusion of other related variables in the regression models (i.e. hypertension, antiplatelet and anticoagulant medication, etc). Presently we do not have a tangible explanation on the observed association of AF and DR, as it also might have been a chance finding. Further studies should aim to detangle the relationship between AF and perfusion outcome.

Previous studies have demonstrated close resemblance in clinical outcome between eTICI 2c and 3 patients [25, 26] and these results were also replicable in our study (Supplementary Table 6). In our cohort 17.2% patients with eTICI 2c showed PPD, meaning that at least a portion of patients (1 out of 5) do not resemble eTICI 3 patients. Of course, the number of eTICI 2c patients included in our study might have reduced power to detect actual difference between eTICI 2c and eTICI 3 patients.

### Potential Role of IVT as Rescue Treatment

While meta-analyses of observational data mostly showed favorable associations of IVT + MT vs. direct MT [3, 33], current synopses of randomized-controlled trials do not support a similar effect [9, 10] potentially because observational data are highly biased by indication [34]. The neutral results of these trials have also sparked the idea that IVT may be administered after incomplete MT (reverse bridging). Moreover, a recent opinion paper listed IVT treatment as a potential factor to consider when planning rescue treatment after incomplete MT [14].

Our analysis did not provide clear evidence for an association of IVT administration and the occurrence of DR on perfusion follow-up imaging. Even when considering different timing strata, adjusted analyses failed to show a significant association. Consequently, these results do not support the consideration on IVT pretreatment as a relevant factor used for the decision-making process on whether to treat or not to treat residual occlusion after incomplete MT.

In our study cohort almost 40% of patients had a PPD. Considering implication of perfusion outcome on functional outcome and mortality rates, PPD patients could constitute an ideal subgroup for pursuing additional rescue treatments; however, at the time point of decision making (after thrombectomy), information on the delayed reperfusion status is not available. Accurate prediction of perfusion outcome may help in the selection of patients for e.g. intra-arterial thrombolysis [35, 36] administration of antithrombotics [37, 38] or secondary distal MT [39, 40]. While potentially beneficial, all of these treatments also harbor a risk of harm, warranting a careful patient selection. Clearly, patients who will have PPD on follow-up imaging may constitute an ideal subgroup where the risk-benefit profile for rescue treatment options seems favorable, while the situation might be less clear in patients who experience DR.

### Limitations

Our study has several limitations. Firstly, we have performed a single-center retrospective data analysis limiting generalizability of our results to other cohorts. Secondly, there was a relevant selection bias, based on the inclusion criteria. Patients who had received follow-up perfusion imaging in the present study had less comorbidities and a more favorable baseline profile. Hence, the absolute number of observed DR should be handled with caution, because we cannot infer that the absolute rates of DR would be the same in patients not undergoing perfusion follow-up imaging. Third, there was a reduced power to detect a potentially beneficial effect of IVT in patients with incomplete reperfusion, because only few patients had IVT timings where at least some therapeutic alteplase concentrations could have been expected when incomplete MT occurred.

## Conclusion

DR at ~24 h occurs in around 60% of patients with incomplete reperfusion after MT and does not seem to occur more often in patients receiving preinterventional IVT. While further data on this topic are needed, these results do not support considering the IVT pretreatment status for the decision-making process regarding whether to treat or not to treat residual occlusions after incomplete MT.

## Supplementary Information

Online Resources, Supplementary Methods
