# Supplementary material for: Association of Intravenous Thrombolysis with Delayed Reperfusion After Incomplete Mechanical Thrombectomy
Source: Clin Neuroradiol. 2022 Jul 14;33(1):87–98. doi: 10.1007/s00062-022-01186-7 (PMC10014807; doi:10.1007/s00062-022-01186-7)
Supplement: Supplementary file 1 — Online Resources, Supplementary Methods [file 62_2022_1186_MOESM1_ESM.pdf]

ONLINE RESOURCES

Association of Intravenous Thrombolysis with Delayed Reperfusion after Incomplete Mechanical Thrombectomy

Table of Contents

- Supplementary Figure I IVT Stratification Sets
- Supplementary Figure II False Positives of Persistent Perfusion Deficit on Perfusion Follow-Up Imaging
- Supplementary Figure III Study Flow Chart
- Supplementary Figure IV Association of Intervention-To-Follow-Up Time with the Probability of a Delayed Reperfusion Occurrence.
- Supplementary Table I Packages Used for Statistical Analysis in R v4.0.0
- Supplementary Table II Comparison of Patients With and Without a Perfusion Follow-Up Imaging
- Supplementary Table III Sensitivity Analysis Including Patients Who Have and Have Not Received Intravenous Thrombolysis
- Supplementary Table IV Output of the Logistic Regression Model Sensitivity Analyses With Delayed Reperfusion as a Depended Variable
- Supplementary Table V Patients with Delayed Reperfusion and Persistent Perfusion Deficit of the Affected Tissue Stratified by Different Intravenous Thrombolysis Time Windows
- Supplementary Table VI Functional Outcome and Mortality Rates at Three Months With Delayed Reperfusion as Variable of Interest

Supplementary Figure I IVT Stratification Sets

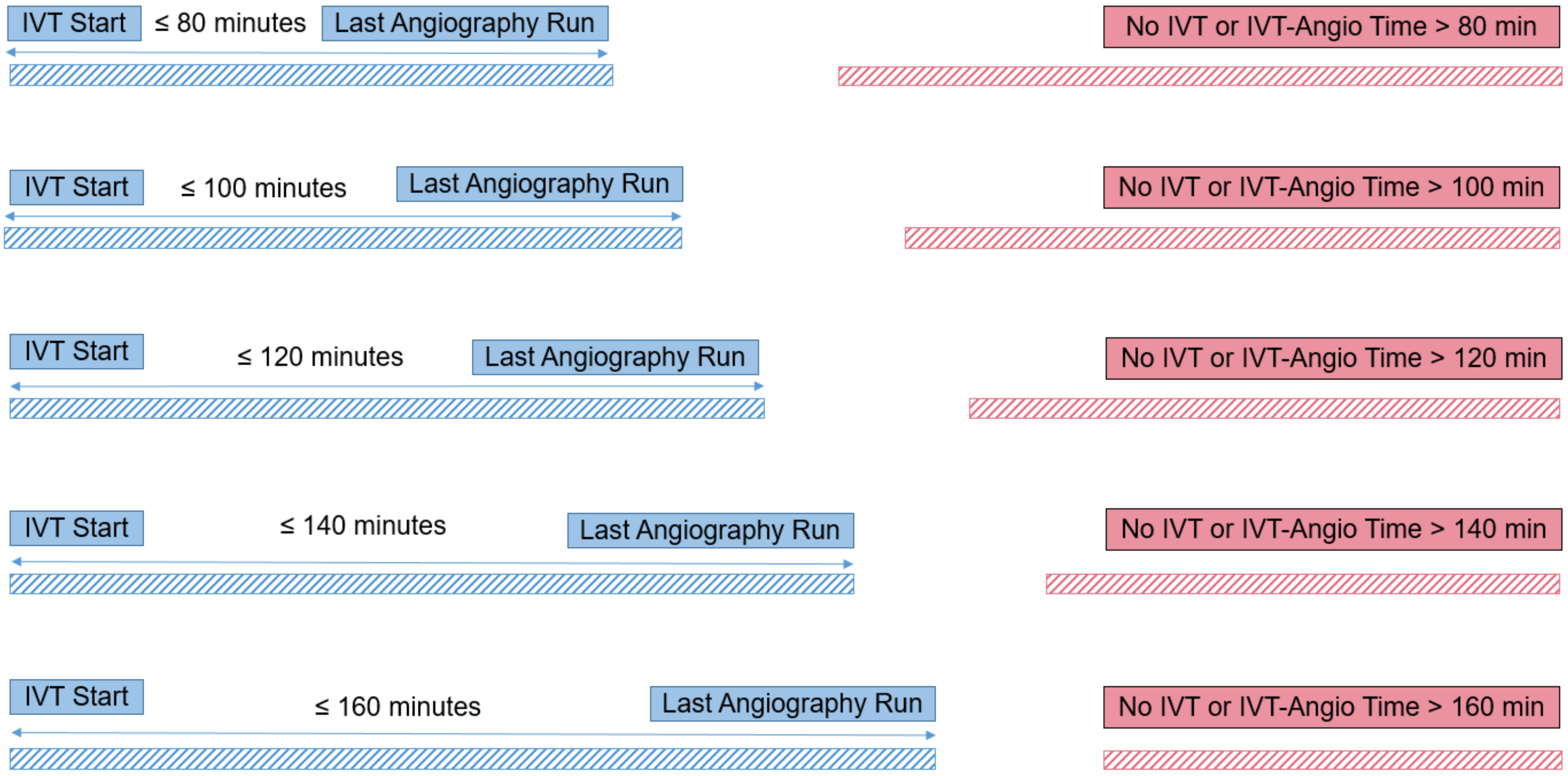

IVT: intravenous thrombolysis. Additional sensitivity analysis dichotomized all patients into either of the two subgroups: (I) patients with short intervals between IVT start and the occurrence of incomplete MT as seen on the final angiography imaging vs (II) patients with long intervals between IVT start and the occurrence of incomplete MT as seen on the final angiography imaging. For example, using an 80 minute cut-off based on atleplase terminal half-life, we compared all patients who had received IVT within 80 minutes before the occurrence of incomplete MT was noted on the final angiography run, versus patients who have passed the 80 minute threshold from reciving IVT to the occurrence of incomplete MT on the final angiography run. The rationale behind this dichotomization was that patients with shorter time intervals between IVT start until the occurrence of incomplete MT were more likely to have therapeutic concentrations of circulating alteplase, while those with longer intervals between IVT start and the occurrence of incomplete MT were less likely to have it. In total, there were 5 sets for 80, 100, 120, 140 and 160-minute time windows

Supplementary Figure II False Positives of Persistent Perfusion Deficit on Perfusion Follow-Up Imaging

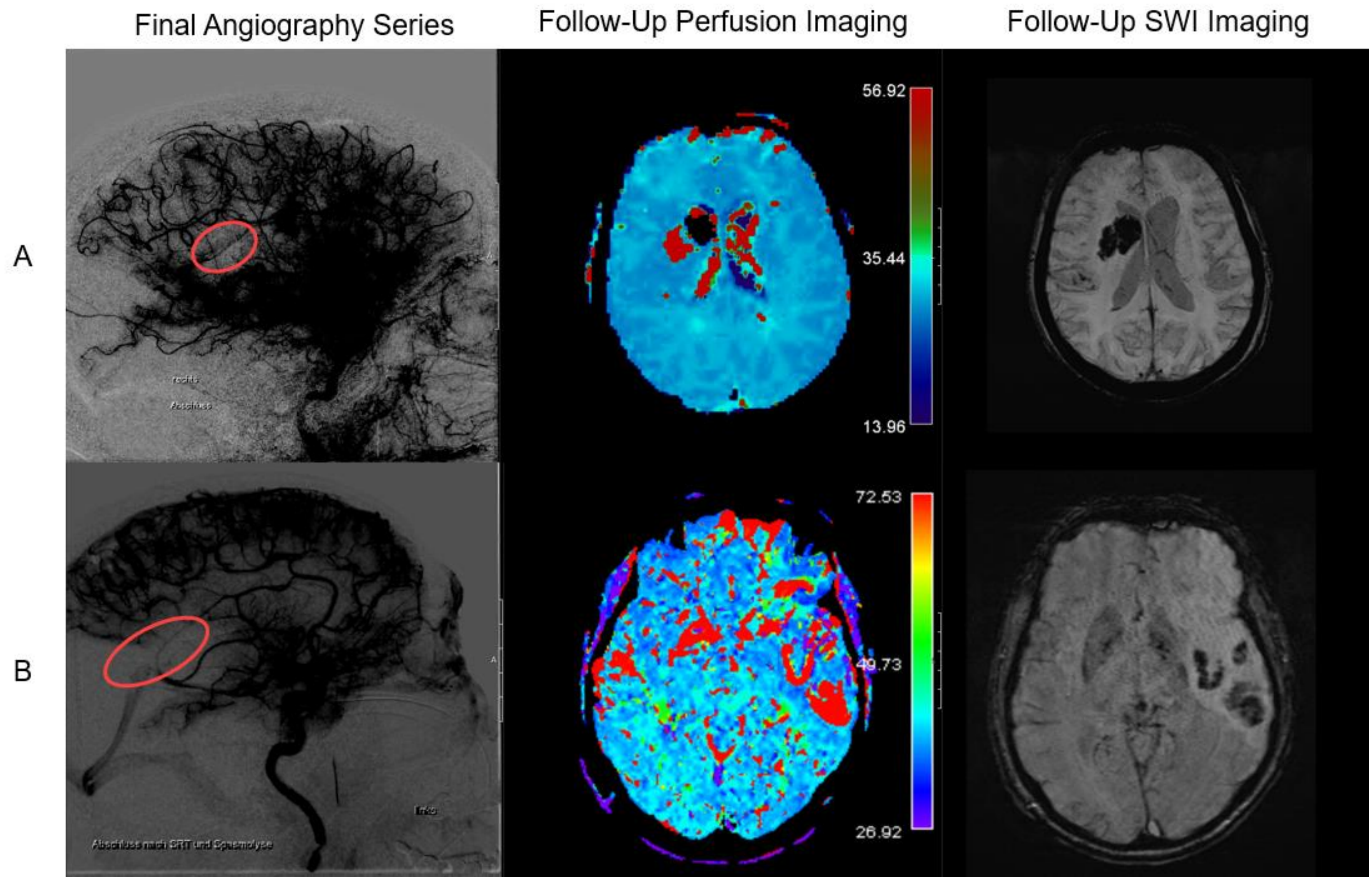

Patient which could be classified as having a persistent perfusion deficit after having a (A) right side M1 occlusion and achieving eTICI 2b50 reperfusion; or (B) left side M1 occlusion and achieving eTICI 2b50 reperfusion. TTP and Tmax perfusion imaging maps were evaluated on follow up examinations due to their high sensitivity rates (Olea Sphere v2.3; Olea Medical & syngo.via, Siemens). Upon additional examination on MRI susceptibility-weighted imaging we can conclude that the non-reperfused territory has already underwent hemorrhagic transformation and does not represent a true persistent perfusion deficit.

Supplementary Figure III Study Flow Chart

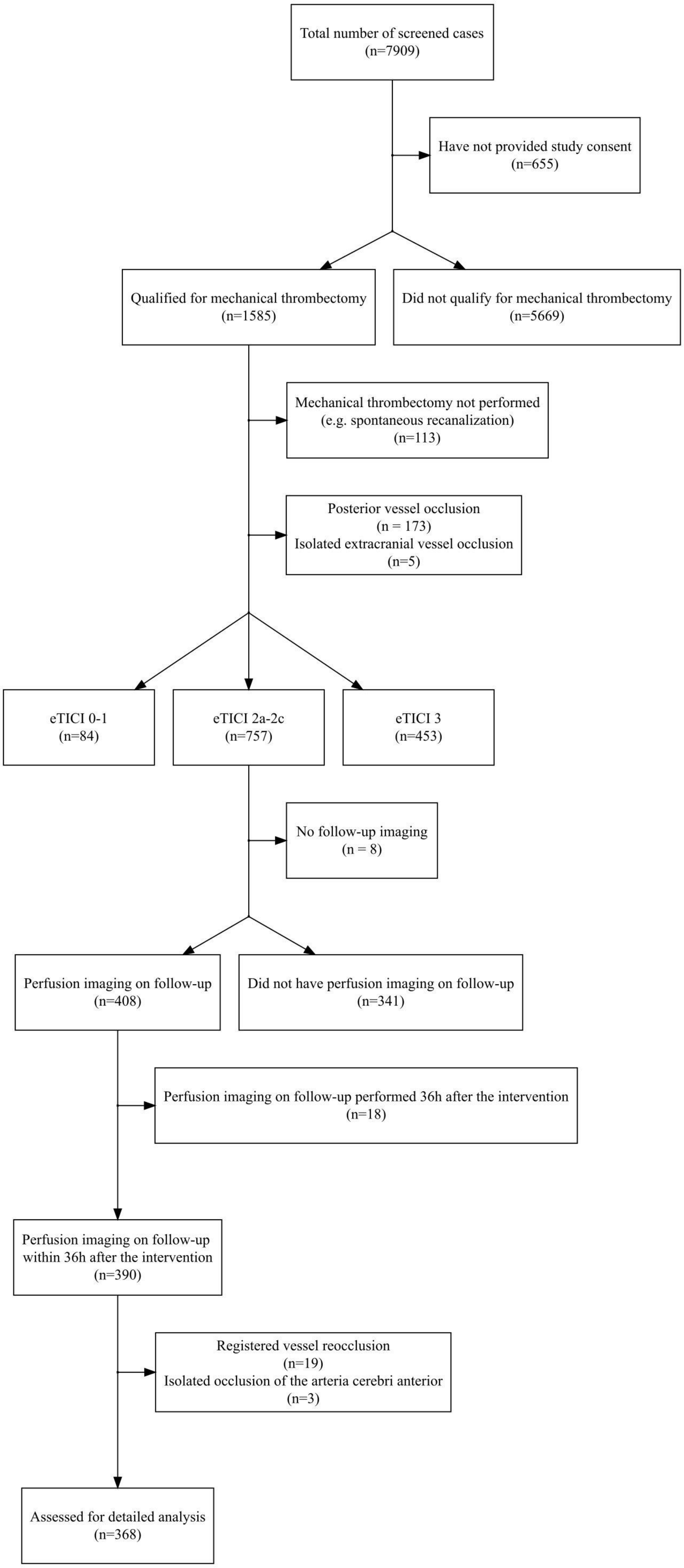

Supplementary Figure IV Association of Intervention-To-Follow-Up Time with the Probability of a Delayed Reperfusion Occurrence.

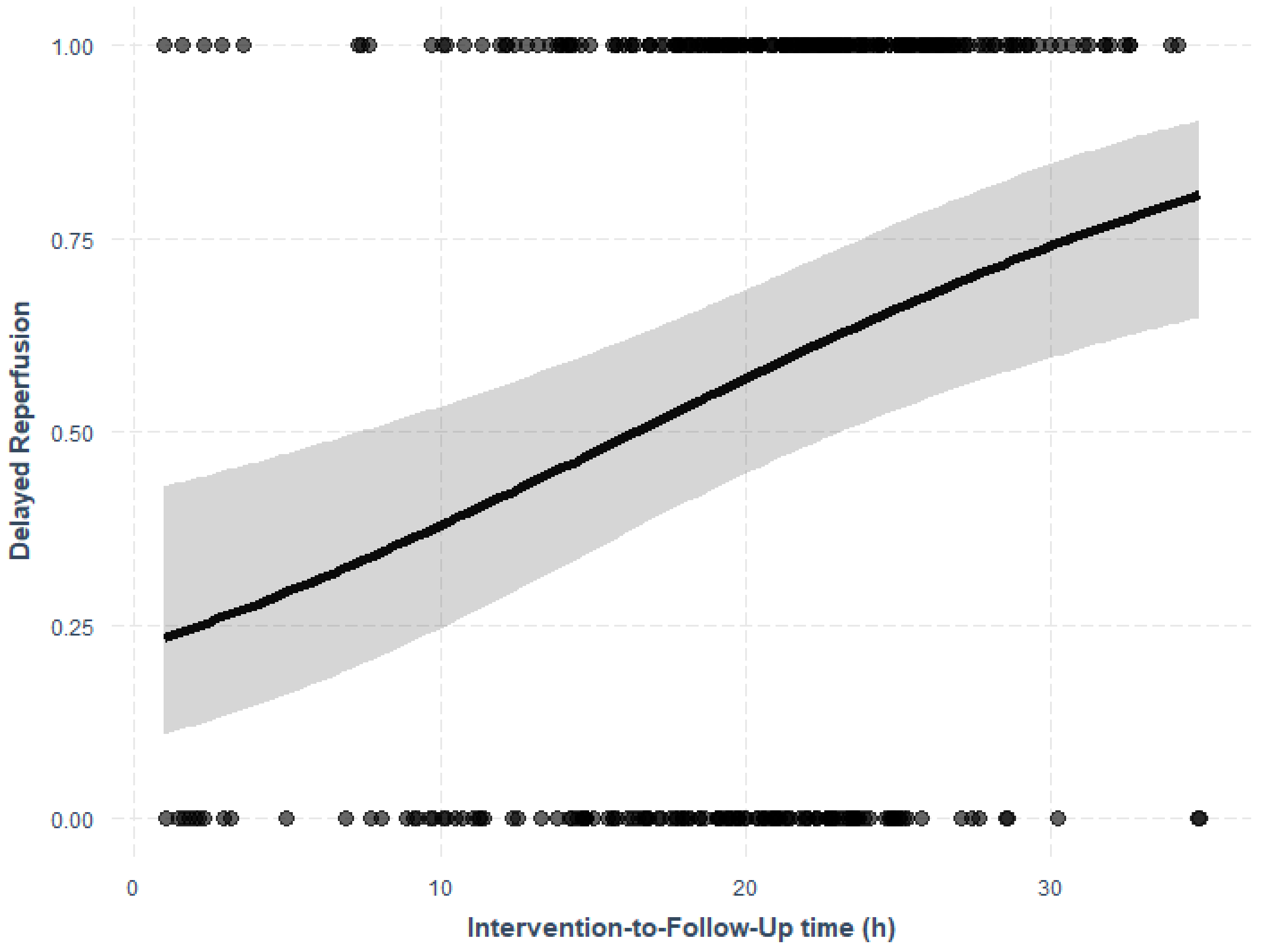

Adjusted predicted probability plot of delayed reperfusion according to the intervention-to-follow-up time in hours. A statistically strong association is found between intervention-to-follow-up time and status of delayed reperfusion (aOR 1.08 [95% CI 1.04 - 1.13] per hour delay; p <0.001), while holding all other variables constant (see Methodology).

Supplementary Table I Packages Used for Statistical Analysis in R v4.0.0

**Package Name**	**Version**
Tables	
readxl	1.3.1
tidyverse	1.3.1
ggplot2	3.3.4
tibble	3.1.2
tidyr	1.1.3
readr	1.4.0
purrr	0.3.4
dplyr	1.0.6
stringr	1.4.0
forcats	0.5.1
tableone	0.12.0
Logistic Regression Models	
sjPlot	2.8.8
questionr	0.7.4
MuMIn	1.43.17
Predicated Probability Model	
jtools	2.1.3
Kaplan-Meier Curves	
survival	3.1-12
ggplot2	3.3.4
ggpubr	0.4.0
survminer	0.4.9
flexsurv	2.0
lattice	0.20-41
Formula	1.2-4
Hmisc	4.5-0
SparseM	1.81
rms	6.2-0
jskm	0.4.2
survRM2	1.0-3
bibtex	0.4.2.3
hutils	1.6.0
Modified Rankin Scale Plots	
gridExtra	2.3
Forest Plots	
sjPlot	2.8.8
jtools	2.1.3
ggstance	0.3.5
RColorBrewer	1.1-2

Supplementary Table II Comparison of Patients With and Without a Perfusion Follow-Up Imaging

	Missing values	Overall	Perfusion	No perfusion	p
**n**		744	405	339	
BASELINE					
Age		75.15 [63.2, 83.2]	73.6 [61.7, 80.7]	77.1 [64.8, 85.2]	<0.001
Sex (female)		373 (50.1)	202 (49.9)	171 (50.4)	0.936
MEDICAL HISTORY					
Atrial fibrillation		279 (37.5)	136 (33.6)	143 (42.2)	0.019
Coronary heart disease		139 (18.7)	70 (17.3)	69 (20.4)	0.329
Diabetes		119 (16.0)	61 (15.1)	58 (17.1)	0.510
Hyperlipidemia		451 (60.6)	259 (64.0)	192 (56.6)	0.050
Hypertension		539 (72.0)	277 (68.4)	259 (76.4)	0.019
Intracranial hemorrhage		9 (1.2)	6 (1.5)	3 (0.9)	0.686
Smoking status		158 (21.2)	82 (20.2)	76 (22.4)	0.528
Previous stroke		94 (12.6)	43 (10.6)	51 (15.0)	0.089
Previous TIA		33 (4.4)	19 (4.7)	14 (4.1)	0.848
Prosthetic heart valve					0.385
Biological		17 (2.3)	9 (2.2)	8 (2.3)	
Mechanical		13 (1.7)	5 (1.2)	8 (2.3)	
None		719 (96.0)	394 (96.6)	325 (95.3)	
Systolic blood pressure on admission (mmHg)		154 [135, 174]	150 [132, 170]	159 [137, 177]	0.001
Diastolic blood pressure on admission (mmHg)		80 [70, 95]	80 [70, 93]	84 [68, 99]	0.078
Creatinine on admission (umol/L)	1 (0.1)	80 [65, 94]	78 [65, 92]	81 [66, 96]	0.100
Glucose on admission (mmol/L)	3 (0.4)	6.6 [5.8, 8]	6.5 [5.8, 8]	6.7 [5.8, 8.1]	0.213
Anticoagulants pre-stroke	1 (0.1)	127 (17.1)	58 (14.3)	69 (20.4)	0.049
Antiplatelets pre-stroke	2 (0.3)	217 (29.2)	113 (27.9)	104 (30.7)	0.700
mRS 0-1 pre-stroke		560 (75.3)	327 (80.7)	233 (68.7)	<0.001
NIHSS on admission		14 [8, 19]	13 [8, 19]	16 [9, 20]	0.004
Known symptom onset					0.899
no		192 (25.8)	104 (25.7)	88 (26.0)	
wake up		137 (18.4)	77 (19.0)	60 (17.7)	
yes		415 (55.8)	224 (55.3)	191 (56.3)	
INTERVENTION					
Onset-To-Door (h)	23 (3.1)	3.2 [1.7, 7.2]	3.2 [1.8, 7.2]	3.2 [1.6, 7.3]	0.889
Intravenous Thrombolysis		256 (34.4)	144 (35.6)	112 (33.0)	0.521
NEUROIMAGING					
Occlusion sites					0.051
ICA		176 (23.7)	80 (19.8)	96 (28.3)	
M1		371 (49.9)	215 (53.1)	156 (46.0)	
M2		185 (24.9)	104 (25.7)	81 (23.9)	
M3		12 (1.6)	6 (1.5)	6 (1.8)	
eTICI					0.049
2a		69 (9.3)	28 (6.9)	41 (12.1)	
2b50		116 (15.6)	58 (14.3)	58 (17.1)	
2b67		291 (39.1)	165 (40.7)	126 (37.2)	
2c		268 (36.0)	154 (38.0)	114 (33.6)	
OUTCOME					
mRS at 90-days	1 (0.1)	3 [1, 5]	2 [1, 5]	4 [2, 6]	<0.001

Categorical data are expressed as real numbers (n) and percentages (%). Continuous data are presented as median (n) and interquartile range [IQR]. TIA: transitory ischemic attack; mRS: modified Rankin Scale; NIHSS: National Institutes of Health Stroke Scale; ICA: Intracracnial Carotid Artery Occlusion; eTICI: extended Thrombolysis in Cerebral Infarction; M1: Proximal Occlusion of the Middle Cerebral Artery; M2: Sylvian Segment Occlusion of the Middle Cerebral Artery; M3: Cortical Segment Occlusion of the Middle Cerebral Artery; A1-2: Pre-Communicating and Post-Communicating Segment Occlusion of the Anterior Cerebral Artery.

Supplementary Table III Sensitivity Analysis Including Patients Who Have and Have Not Received Intravenous Thrombolysis

	**Delayed Reperfusion**						
	**Patients who have received IVT**				**Patients who have not received IVT**		
Predictors	Adjusted Odds Ratios	95% CI	P-Value		Adjusted Odds Ratios	95% CI	P-Value
Age	0.99	0.95 – 1.02	0.545		0.98	0.94 – 1.01	0.141
Sex	1.66	0.62 – 4.70	0.323		1.36	0.67 – 2.77	0.393
Atrial Fibrillation	6.97	1.81 – 35.71	0.009		1.48	0.64 – 3.47	0.357
Anticoagulants pre-stroke	0.14	0.01 – 1.94	0.133		1.03	0.40 – 2.68	0.953
Antiplatelets pre-stroke	1.10	0.30 – 4.14	0.879		0.64	0.30 – 1.36	0.243
NIHSS on Admission	1.00	0.94 – 1.07	0.942		1.01	0.96 – 1.06	0.657
Onset-to-Door (h)	1.04	0.94 – 1.24	0.594		1.00	0.95 – 1.06	0.975
Intervention-to-Follow-Up (h)	1.08	1.00 – 1.18	0.053		1.08	1.03 – 1.15	0.004
Device passes	0.89	0.59 – 1.34	0.575		0.91	0.70 – 1.18	0.471
Collaterals	1.78	1.08 – 3.16	0.032		1.50	1.09 – 2.08	0.014
eTICI 2b50	0.16	0.01 – 3.21	0.214		1.53	0.27 – 12.20	0.649
eTICI 2b67	5.15	0.47 – 86.16	0.203		7.73	1.74 – 54.98	0.015
eTICI 2c	22.62	1.96 – 425.00	0.019		25.21	5.31 – 188.77	<0.001
ΔR^2m	0.512				0.338		

NIHSS: National Institutes of Health Stroke Scale; eTICI: extended Thrombolysis in Cerebral Infarction. Adjusted odds ratios (aOR) for variable “Age” refer to 10-year increase and aOR for variable “NIHSS on Admission” to a 5-point increase.

Supplementary Table IV Output of the Logistic Regression Model Sensitivity Analyses With Delayed Reperfusion as a Depended Variable

	**Delayed Reperfusion**						
	**eTICI 2a-2c patients with reoclussions being classified as PPD**				**eTICI 2a-2b67 patients with reoclussions being classified as PPD**		
Predictors	Adjusted Odds Ratios	95% CI	P-Value		Adjusted Odds Ratios	95% CI	P-Value
Age	0.83	0.66 – 1.04	0.105		0.73	0.54 – 0.97	0.033
Sex	1.47	0.85 – 2.56	0.174		1.44	0.72 – 2.92	0.301
Atrial Fibrillation	2.43	1.25 – 4.85	0.010		2.75	1.23 – 6.32	0.015
Anticoagulants pre-stroke	0.78	0.32 – 1.92	0.583		1.43	0.49 – 4.22	0.514
Antiplatelets pre-stroke	0.86	0.46 – 1.60	0.631		0.85	0.38 – 1.87	0.680
NIHSS on Admission	1.06	0.88 – 1.28	0.517		1.05	0.83 – 1.32	0.688
Onset-to-Door (h)	1.00	0.96 – 1.05	0.951		0.97	0.91 – 1.04	0.418
Intervention-to-Follow-Up (h)	1.08	1.04 – 1.13	<0.001		1.10	1.04 – 1.17	0.001
Thrombolysis	0.80	0.44 – 1.46	0.462		0.68	0.31 – 1.46	0.323
Device passes	0.87	0.70 – 1.08	0.217		0.92	0.71 – 1.19	0.555
Collaterals	1.59	1.23 – 2.09	0.001		1.78	1.27 – 2.54	0.001
eTICI 2b50	0.84	0.20 – 4.00	0.813		0.87	0.20 – 4.28	0.853
eTICI 2b67	5.57	1.63 – 23.54	0.010		6.46	1.80 – 28.44	0.007
eTICI 2c	21.63	5.95 – 96.69	<0.001				
ΔR^2m	0.358				0.362		

NIHSS: National Institutes of Health Stroke Scale; eTICI: extended Thrombolysis in Cerebral Infarction; PPD: Persistent perfusion deficit. Adjusted odds ratios (aOR) for variable “Age” refer to 10-year increase and aOR for variable “NIHSS on Admission” to a 5-point increase.

Supplementary Table V Patients with Delayed Reperfusion and Persistent Perfusion Deficit of the Affected Tissue Stratified by Different Intravenous Thrombolysis Time Windows

	**Strata Set**	**Delayed Reperfusion**	**Persistent Perfusion Deficit**	**Unadjusted**		**Adjusted**	
				OR	p	aOR	p
n		225	143				
IVT - 80 min	IVT ≤ 80 min	25 (30.9)	7 (13.0)	2.99 [1.24 – 8.07]	0.019	2.28 [0.65 – 9.23]	0.218
	IVT > 80 min	56 (69.1)	47 (87.0)				
IVT - 100 min	IVT ≤ 100 min	34 (42.5)	11 (20.4)	2.88 [1.33 – 6.63]	0.009	1.53 [0.52 – 4.73]	0.442
	IVT > 100 min	46 (57.5)	43 (79.6)				
IVT - 120 min	IVT ≤ 120 min	39 (48.1)	14 (25.9)	2.65 [1.28 – 5.74]	0.011	2.14 [0.76 – 6.41]	0.159
	IVT > 120 min	42 (51.9)	40 (74.1)				
IVT - 140 min	IVT ≤ 140 min	43 (51.1)	22 (40.7)	1.65 [0.82 – 3.33]	0.161	1.31 [0.50 – 3.48]	0.578
	IVT > 140 min	38 (46.9)	32 (59.3)				
IVT - 160 min	IVT ≤ 160 min	44 (55.0)	23 (45.6)	1.65 [0.82 – 3.33]	0.160	1.45 [0.55 – 3.87]	0.455
	IVT > 160 min	36 (45.0)	31 (57.4)				

IVT: intravenous thrombolysis; OR: odds ratios; aOR: adjusted odds ratios.

Supplementary Table VI Functional Outcome and Mortality Rates at Three Months With Delayed Reperfusion as Variable of Interest

	**Delayed Reperfusion**							
Predictors	aOR	95% CI	Predictors	aOR	95% CI	Predictors	aOR	95% CI
**90-Day mRS 0-1**	1.80	1.01 – 3.37	**90-Day mRS 0-2**	2.50	1.35 – 4.71	**90-Day Mortality**	0.70	0.31 – 1.60
Age	0.98	0.96 – 1.01		0.99	0.96 – 1.01		0.98	0.96 – 1.01
Sex	1.50	0.86 – 2.62		1.47	0.85 – 2.59		1.18	0.66 – 2.12
Atrial Fibrillation	2.60	1.32 – 5.26		2.81	1.41 – 5.78		2.20	1.09 – 4.57
Anticoagulants pre-stroke	0.78	0.32 – 1.92		0.74	0.30 – 1.84		0.77	0.30 – 1.98
Antiplatelets pre-stroke	0.88	0.47 – 1.66		0.91	0.48 – 1.73		0.95	0.50 – 1.85
NIHSS on Admission	1.03	0.99 – 1.07		1.03	0.99 – 1.08		1.02	0.98 – 1.06
Onset-to-Door (h)	1.00	0.96 – 1.06		1.01	0.96 – 1.06		1.02	0.97 – 1.07
Intervention-to-Follow-Up (h)	1.08	1.03 – 1.13		1.07	1.03 – 1.12		1.09	1.04 – 1.14
Thrombolysis	0.84	0.46 – 1.55		0.79	0.42 – 1.46		0.80	0.41 – 1.54
Device passes	0.89	0.72 – 1.11		0.89	0.72 – 1.11		0.85	0.66 – 1.09
Collaterals	1.58	1.22 – 2.08		1.55	1.19 – 2.04		1.55	1.18 – 2.06
eTICI 2b50	0.87	0.20 – 4.32		0.86	0.20 – 4.26		0.73	0.17 – 3.61
eTICI 2b67	5.33	1.50 – 23.37		5.08	1.41 – 22.41		5.54	1.57 – 23.74
eTICI 2c	20.51	5.46 – 94.89		20.22	5.34 – 94.16		17.80	4.77 – 80.57
ΔR^2m	0.372		ΔR^2m	0.387		ΔR^2m	0.355	

NIHSS: National Institutes of Health Stroke Scale; eTICI: extended Thrombolysis in Cerebral Infarction. Adjusted odds ratios (aOR) for variable "Age" refer to 10-year increase and aOR for variable "NIHSS on Admission" to a 5-point increase.

SUPPLEMENT MATERIAL

Association of Intravenous Thrombolysis with Delayed Reperfusion after Incomplete Mechanical Thrombectomy

Supplementary Methods I

Instructions on Evaluation of Perfusion Outcome

Choice of perfusion maps

Perfusion outcome can be evaluated on both CT perfusion (CTP) and MR perfusion (MRP) imaging, by using post-processing perfusion software. We have used syngio.via (Siemens) to process CTP data, and Olea Sphere Software (Olea Sphere v2.3; Olea Medical, La Ciotat, France) to generate MRP maps. For the evaluation of perfusion outcome we have used TTP (Time-to-Peak) and Tmax>4 sec (Time-to-Maximum) maps, due to their high sensitivity.[1–3]

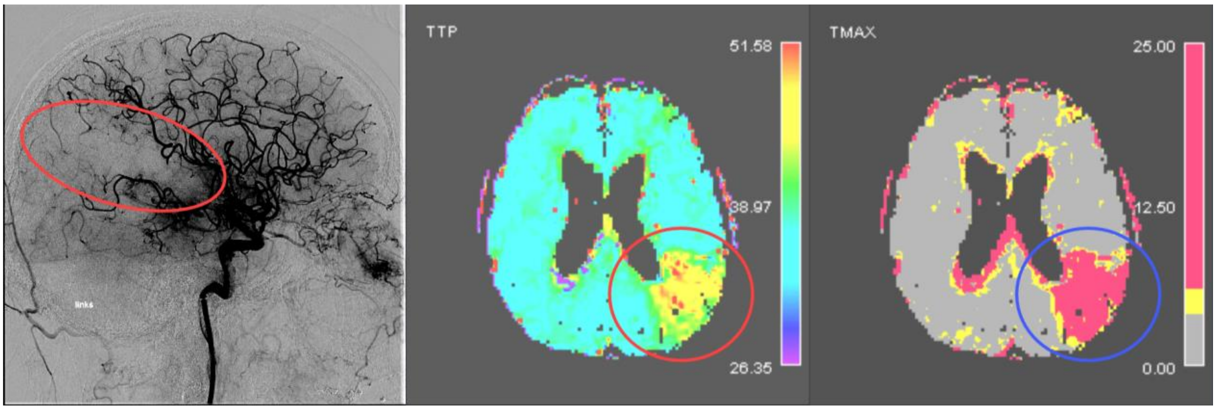

Figure 1. Left: Final angiography imaging with evident incomplete reperfusion. Middle: TTP perfusion deficit which could be classified as a perfusion deficit (red circle). Right: Delay in Tmax indicating the same area of perfusion deficit (blue circle).

Evaluation goal

The goal of the imaging evaluation is to make a statement regarding the persistence or spontaneous reperfusion of distal vessel occlusions visible on post-thrombectomy digital subtraction angiography images. The primary aim is to assess if these distal vessel occlusions are still present on 24h +/-6h follow-up imaging. For this purpose, a combination of pre-interventional cross-sectional imaging, post-thrombectomy digital subtraction angiography imaging, and 24h follow-up imaging was used.

Standardized blinded PACS hanging

For blinded standardized ratings, all evaluated cases were loaded in a working list into the hospital picture archive communicating systems (PACS, Sectra IDS7, Sectra AB, Sweden) using a standardized hanging. All readers are blinded to clinical data during the rating process.

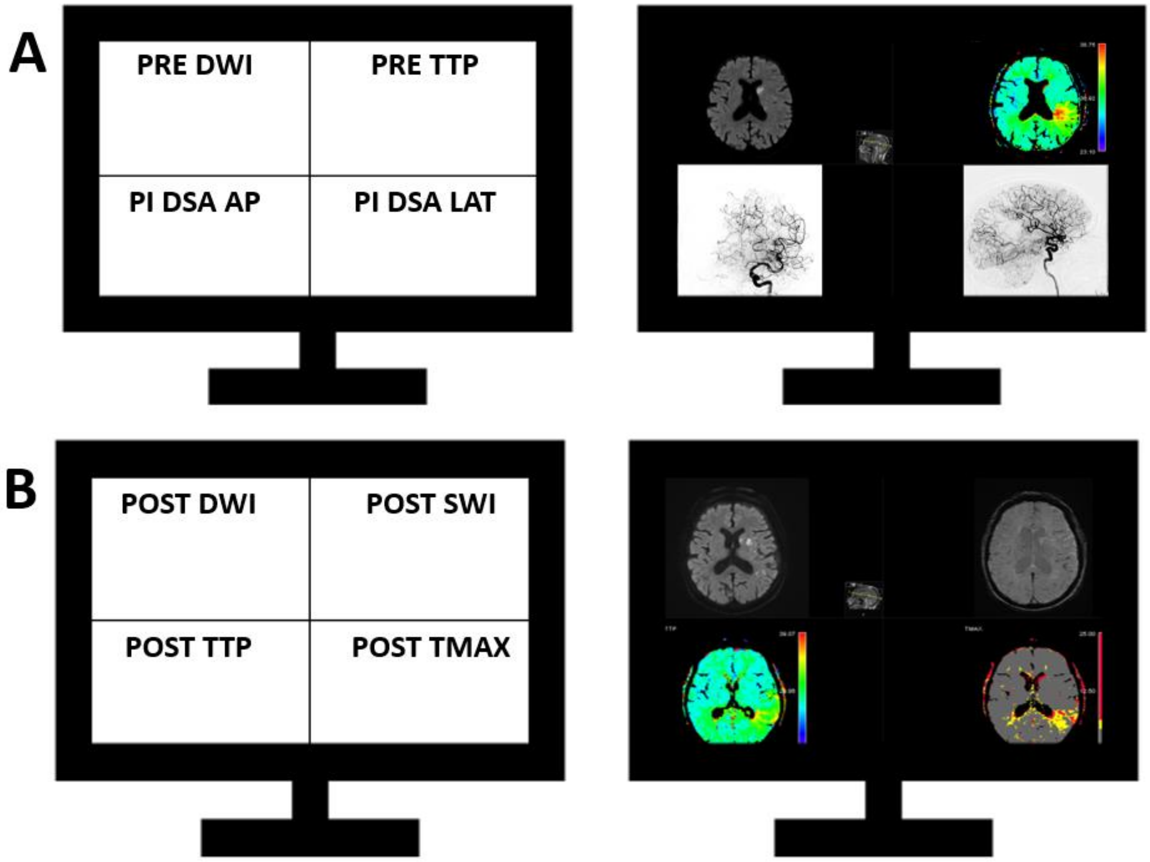

Figure 2A: First hanging panel containing preinterventional diffusion weighted imaging (PRE DWI) and preinterventional time to peak (PRE TTP) map in the top row, together with final perinterventional digital subtraction angiography anteroposterior (PI DSA AP) and lateral (PI DSA LAT) views in the bottom row. Figure 2B: Second hanging panel with postinterventional diffusion weighted imaging (POST DWI) and postinterventional susceptibility weighted imaging (POST SWI) in the top row, together with postintervnetional time-to-peak (POST TTP) and time-to-maximum (POST TMAX) perfusion maps in the bottom row.

Dichotomized perfusion outcomes

The primary imaging outcome is a dichotomized variable indicating either delayed reperfusion or persistent perfusion deficit evaluated on 24-hour follow-up perfusion imaging maps. A persistent perfusion deficit (PPD) was defined as any territorial focal perfusion delay within the initially hypoperfused territory corresponding to the capillary phase deficit on post-thrombectomy digital subtraction angiography images. In a patient with an initial M1-occlusion with a eTICI2b50 reperfusion and a residual M3-branch occlusion would be rated as having a persistent perfusion deficit if Tmax>4 sec, TTP or both maps indicated the presence of a distal vessel occlusion within this area of interest.[4]

- Persistent perfusion deficit

Figure 3. Persistent perfusion deficit with eTICI 2a

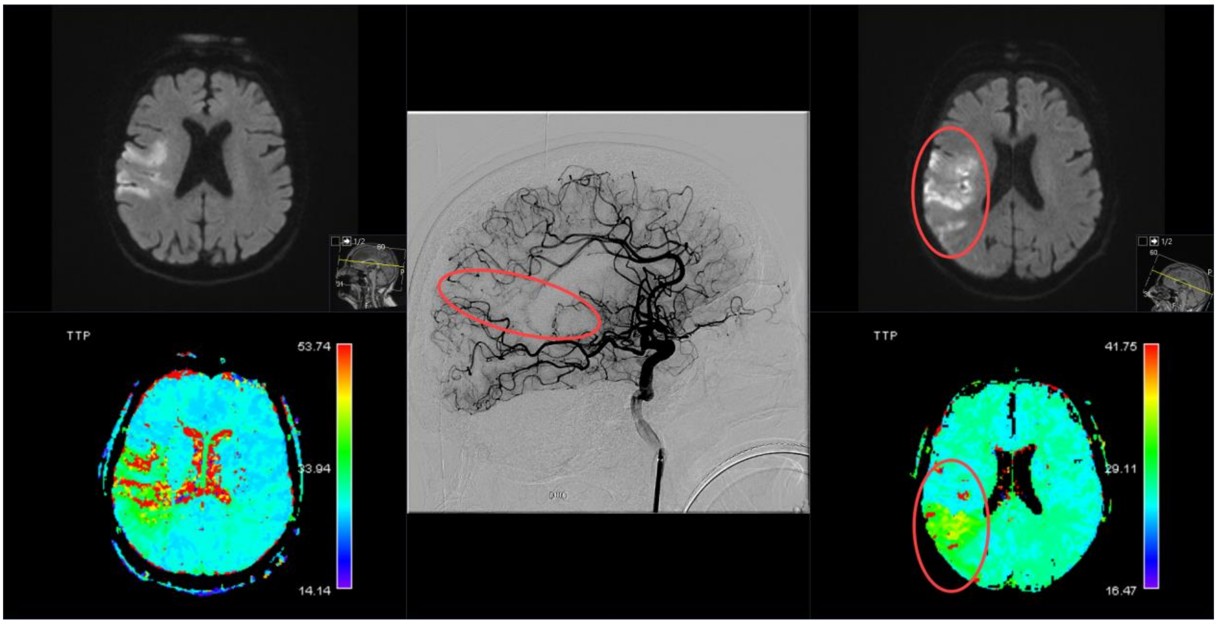

Figure 3. Patient with a right side M2 occlusion with persistent perfusion deficit and confluent-infarct on MRI follow-up after eTICI 2a reperfusion together with corresponding admission DWI (top-left), admission perfusion imaging (bottom-left), final angiography imaging (middle), follow-up DWI (top-right), follow-up perfusion imaging (bottom-right).

Figure 4. Persistent perfusion deficit with eTICI 2b50

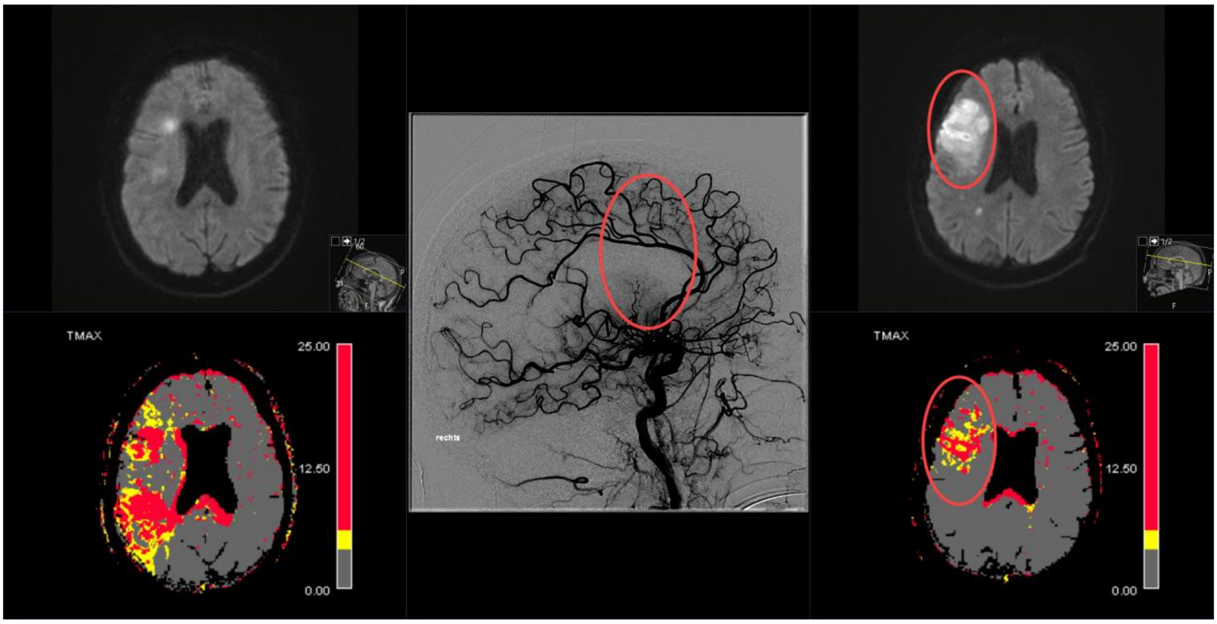

Figure 4. Patient with a left side M2 occlusion with persistent perfusion deficit and multiple emboli type of new infarcts on MRI follow-up after eTICI 2b50 reperfusion together with corresponding admission DWI (top-left), admission perfusion imaging (bottom-left), final angiography imaging (middle), follow-up DWI (top-right), follow-up perfusion imaging (bottom-right).

Figure 5. Persistent perfusion deficit with eTICI 2b67

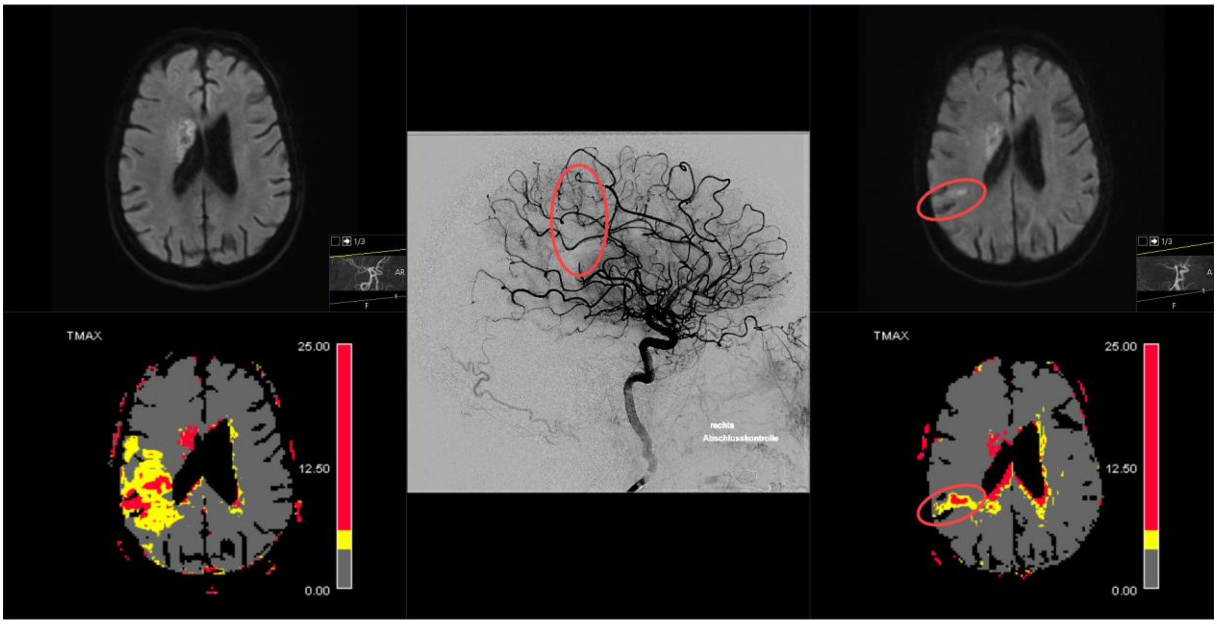

Figure 5. Patient with a left side M2 occlusion with persistent perfusion deficit and emboli type of new infarct on MRI follow-up after eTICI 2b67 reperfusion together with corresponding admission DWI (top-left), admission perfusion imaging (bottom-left), final angiography imaging (middle), follow-up DWI (top-right), follow-up perfusion imaging (bottom-right).

<u>Figure 6. Persistent perfusion deficit with eTICI 2c</u>

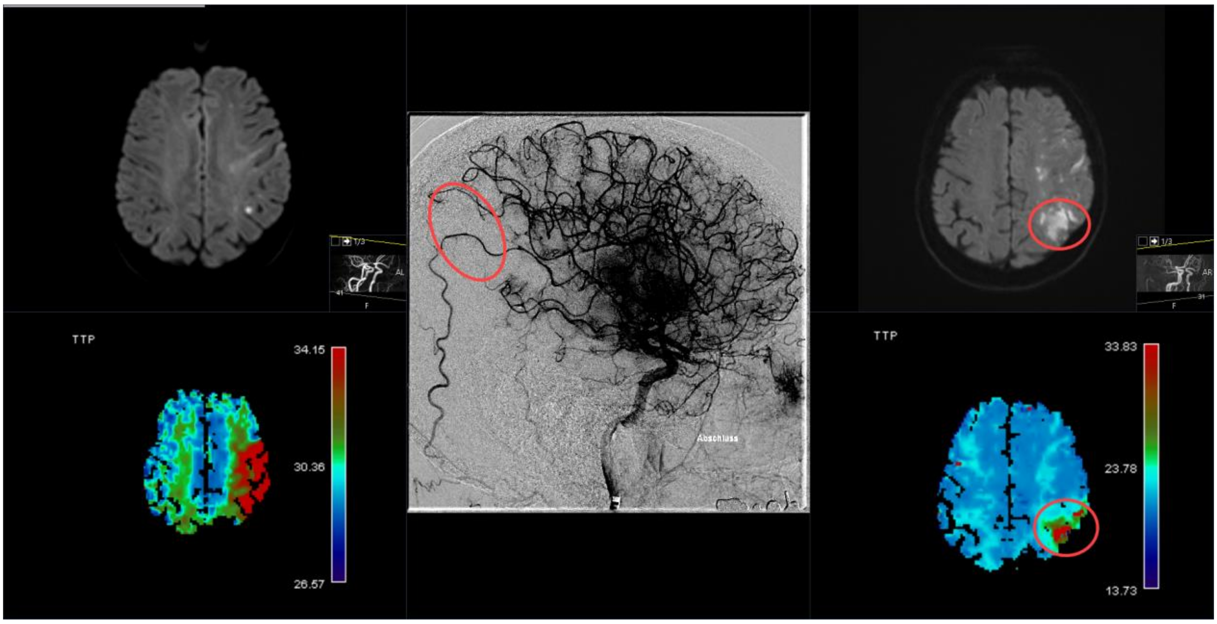

Figure 6. Patient with a left side M2 occlusion with persistent perfusion deficit and confluent-type of new infarct on MRI follow-up after eTICI 2c reperfusion together with corresponding admission DWI (top-left), admission perfusion imaging (bottom-left), final angiography imaging (middle), follow-up DWI (top-right), follow-up perfusion imaging (bottom-right).

- <u>Delayed reperfusion</u>

On the other hand, a delayed reperfusion (DR) is defined as the absence of any territorial focal perfusion delay on TTP, Tmax or both, indicating that there is no residual vessel occlusion.

<u>Figure 7. Delayed reperfusion with 2a</u>

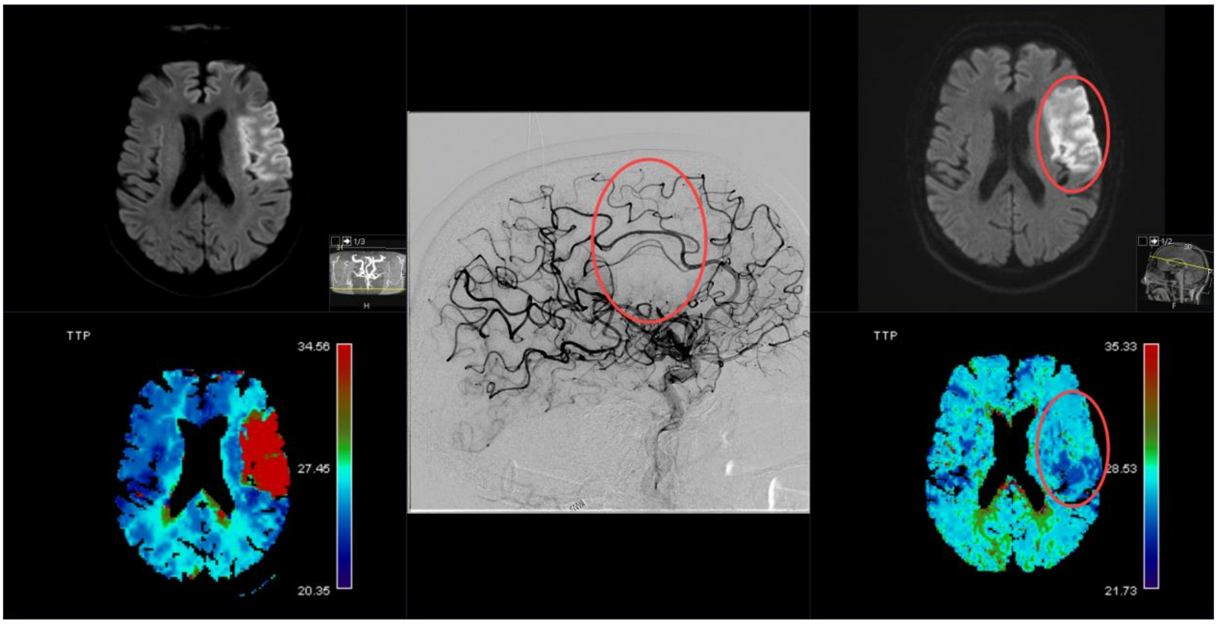

Figure 7. Patient with a left side M1 occlusion with delayed reperfusion and no new infarct on MRI follow-up after eTICI 2a reperfusion together with corresponding admission DWI (top-left), admission perfusion imaging (bottom-left), final angiography imaging (middle), follow-up DWI (top-right), follow-up perfusion imaging (bottom-right).

Figure 8. Delayed reperfusion with 2b50

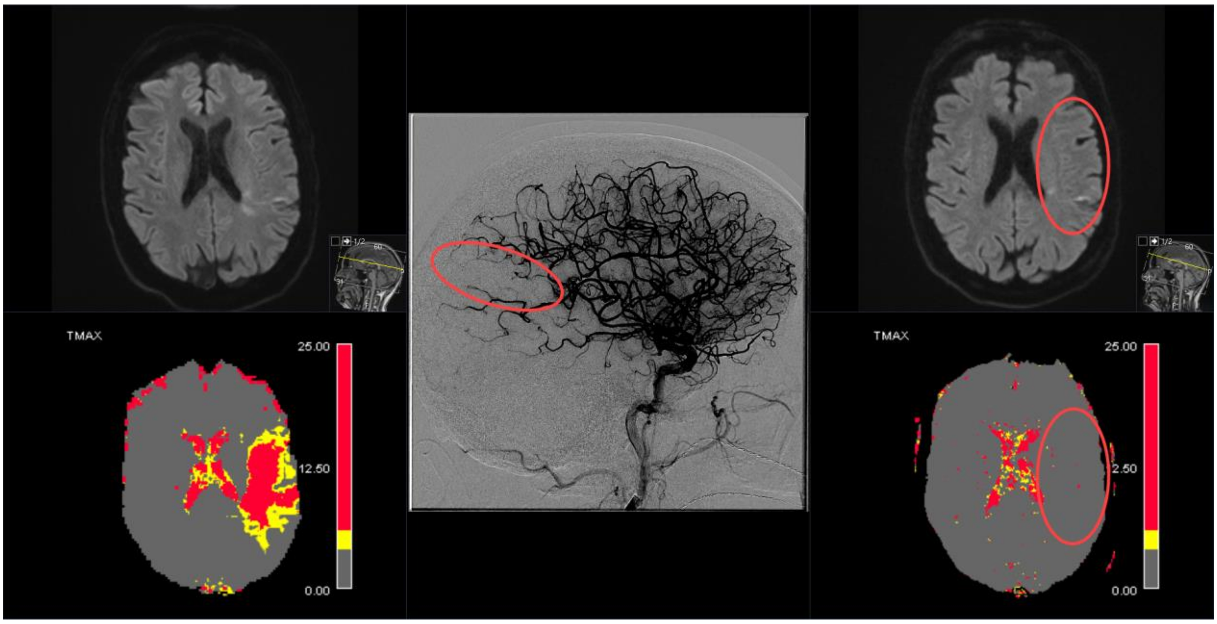

Figure 8. Patient with a right side M1 occlusion with delayed reperfusion and no new infarct on MRI follow-up after eTICI 2b50 reperfusion together with corresponding admission DWI (top-left), admission perfusion imaging (bottom-left), final angiography imaging (middle), follow-up DWI (top-right), follow-up perfusion imaging (bottom-right).

Figure 9. Delayed reperfusion with 2b67

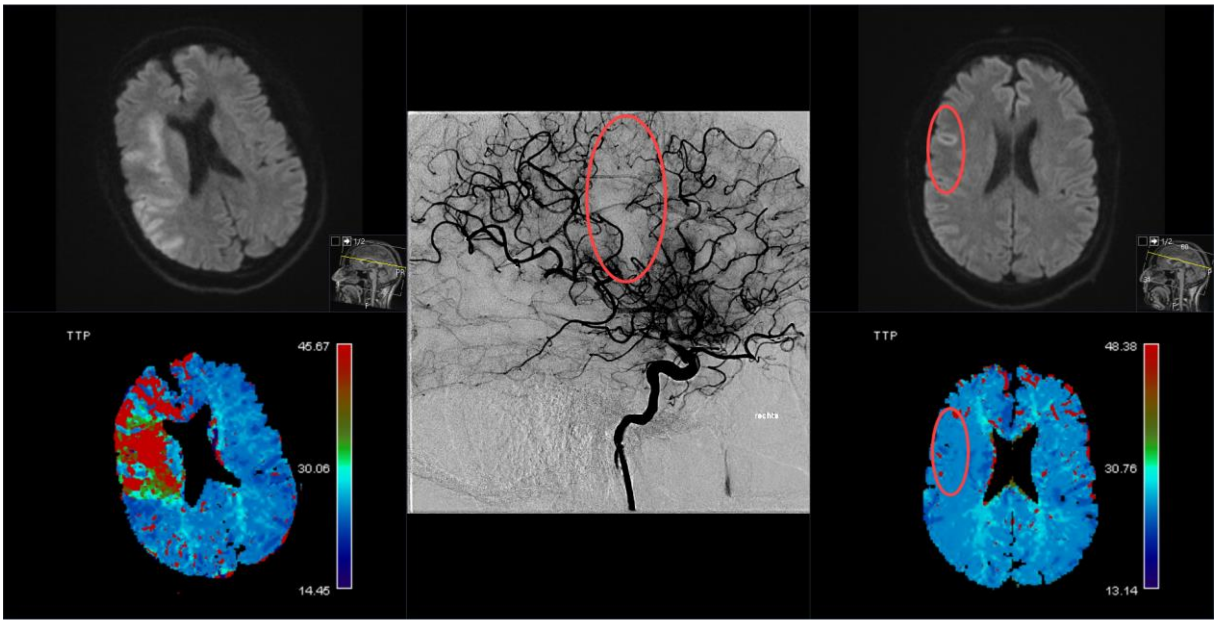

Figure 9. Patient with a right side M1 occlusion with delayed reperfusion and no new infarct on MRI follow-up after eTICI 2b67 reperfusion together with corresponding admission DWI (top-left), admission perfusion imaging (bottom-left), final angiography imaging (middle), follow-up DWI (top-right), follow-up perfusion imaging (bottom-right).

<u>Figure 10. Delayed reperfusion with 2c</u>

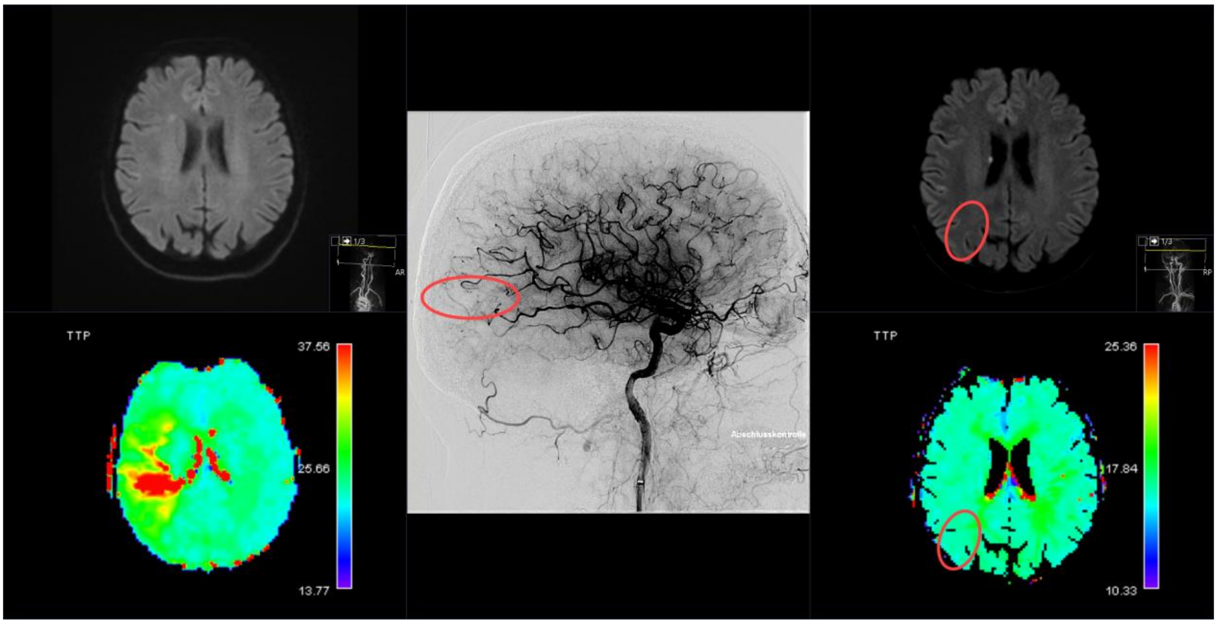

Figure 10. Patient with a right side M1 occlusion with delayed reperfusion and no new infarct on MRI follow-up after eTICI 2c reperfusion together with corresponding admission DWI (top-left), admission perfusion imaging (bottom-left), final angiography imaging (middle), follow-up DWI (top-right), follow-up perfusion imaging (bottom-right).

Special cases:

- **<u>Multiple residual occlusions:</u>**

In cases of multiple residual occlusions after thrombectomy causing incomplete rather than TICI3 reperfusion, delayed reperfusion was only assigned if all distal vessel present on post-thrombectomy DSA images showed spontaneous reperfusion. In a patient with an M1 occlusion and residual M4 occlusion in the parietal and frontal MCA territory and 24h perfusion imaging shows evidence for a persistent occlusion in the parietal MCA territory but reperfusion in the frontal MCA territory, this will be rated as persistent perfusion deficit.

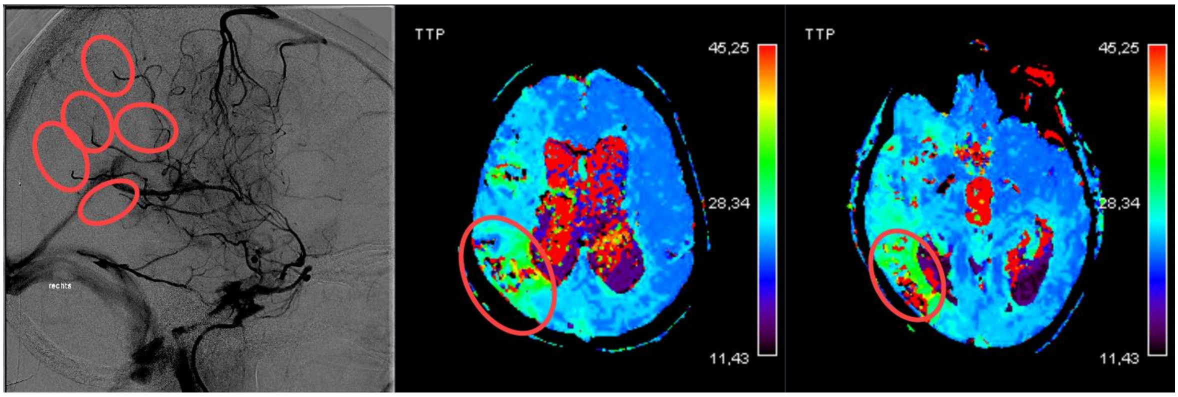

Figure 11. Left panel: Final angiography imaging of a patient with an initial M1 occlusion, showing multiple residual occlusions in distal MCA territory. Right side panels: Showing persistent perfusion deficits on multiple levels, with all of them corresponding to the non-perfused area.

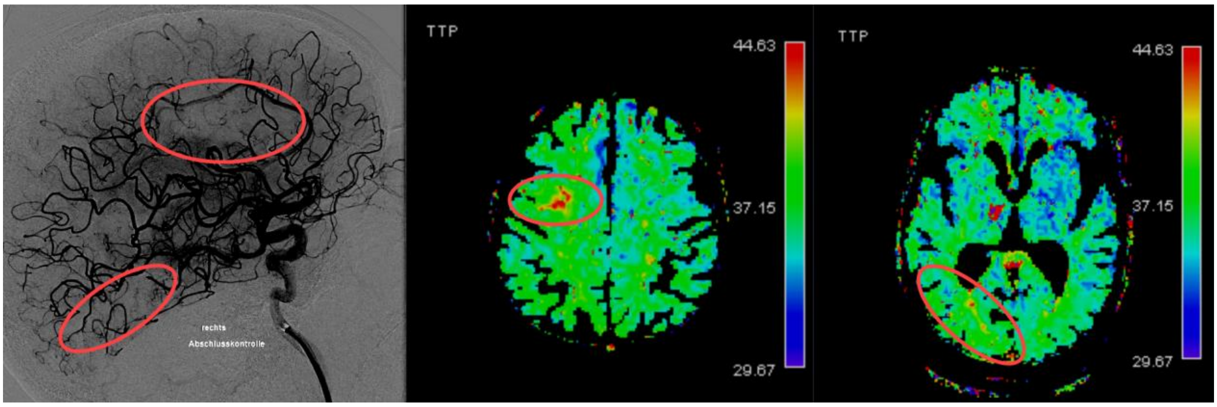

Figure 12. Left panel: Final angiography imaging of a patient with an initial ICA occlusion, showing non-perfused M2 and P3 area. Middle panel: Persistent perfusion deficit observed on follow-up perfusion imaging, directly corresponding to the non-perfused area of distal M2. Right panel: Complete reperfusion corresponding to the P3. This case was rated as persistent perfusion deficit, since not all distal vessel showed spontaneous reperfusion.

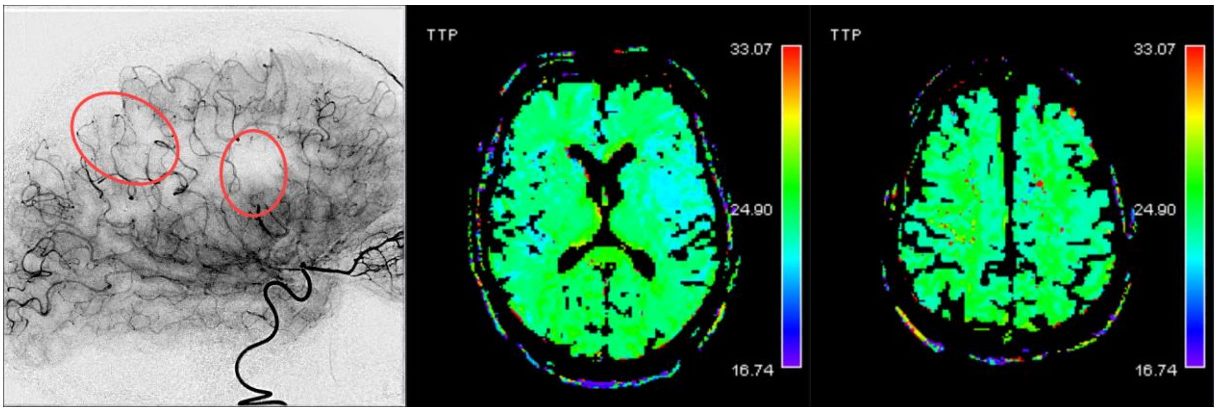

Figure 13. Left panel: Final angiography imaging of a patient with an M1 occlusion, showing non-perfused M2 and M3 areas. Middle and right panel: Delayed reperfusion observed on follow-up perfusion imaging, directly corresponding to the non-perfused area of distal M2 and M3. This case was rated as delayed reperfusion due to all distal vessel having delayed reperfusion.

- **<u>Territorial hemorrhagic transformation</u>**

Blood products cause local distortion of the magnetic field homogeneity leading to loss of signal in the transformed area of interest. On TTP and Tmax maps this will cause a severe delay appearing as a contrast to the surrounding tissue, without a gradual delay cascade.[5] In order to exclude additional bleedings and hemorrhagic tissue transformation, we have used susceptibility weighted imaging (SWI).

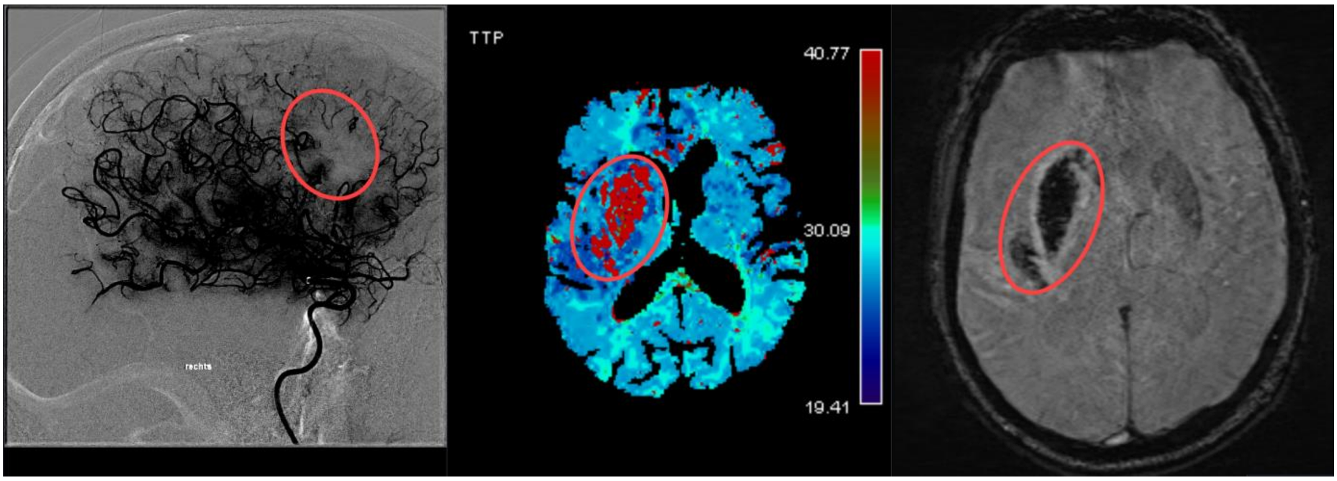

Figure 14. Left panel: Final angiography imaging showing non-complete perfusion. Middle panel: Perfusion anomaly corresponding to the non-perfused area on the final angiography imaging. Right panel: SWI identifies perfusion anomaly as haemorrhagically transformed tissue and not as true PPD.

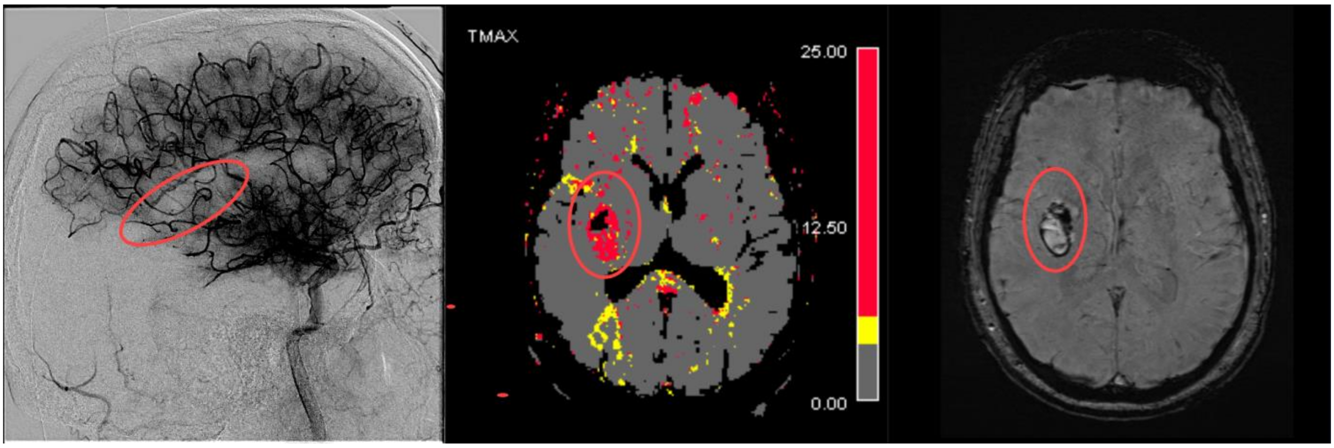

Figure 15. Left panel: Final angiography imaging showing non-complete perfusion. Middle panel: Perfusion anomaly corresponding to the non-perfused area on the final angiography imaging. Right panel: SWI identifies perfusion anomaly as haemorrhagically transformed tissue and not as true PPD.

It is worth noting that bleeding and PPD are not mutually exclusive, warranting an ever greater need in careful interpretation of possible PPD.

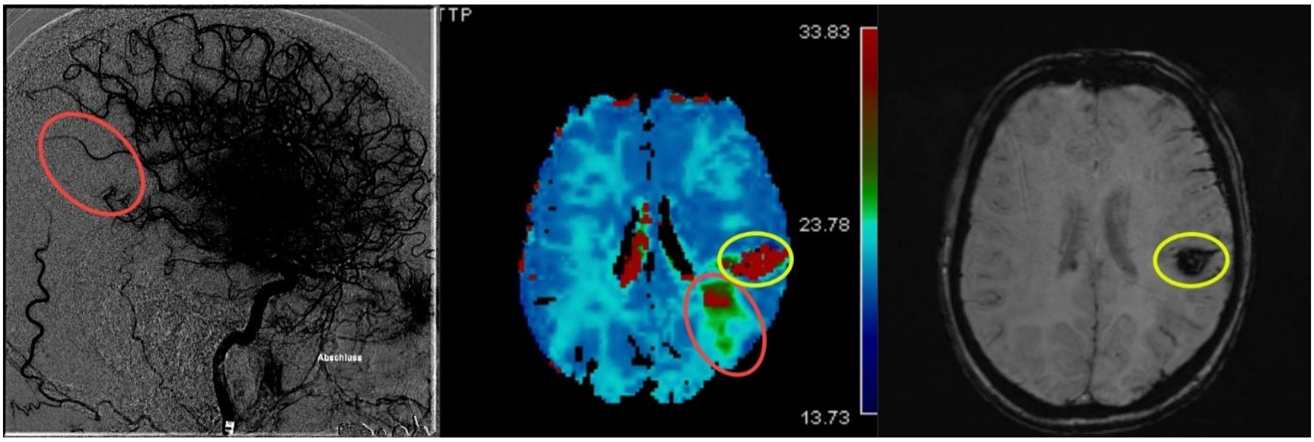

Figure 16. Left panel: Final angiography imaging showing non-complete reperfusion. Middle panel: Perfusion anomalies (red circle) and hemorrhagic transformation (yellow circle) corresponding to the same place as the area of incomplete reperfusion. Right panel: SWI imaging showing that not all anomalies captured on perfusion imaging represent a real PPD.

- **<u>Proximal reocclusion</u>**

Reocclusions were defined as new intracranial occlusions within the same arterial segment which was recanalized at the end of the procedure. They would appear as same perfusion deficit patterns on both pre- and post-treatment perfusion images, that could otherwise not be correlated to the area of interest from the final angiography imaging.

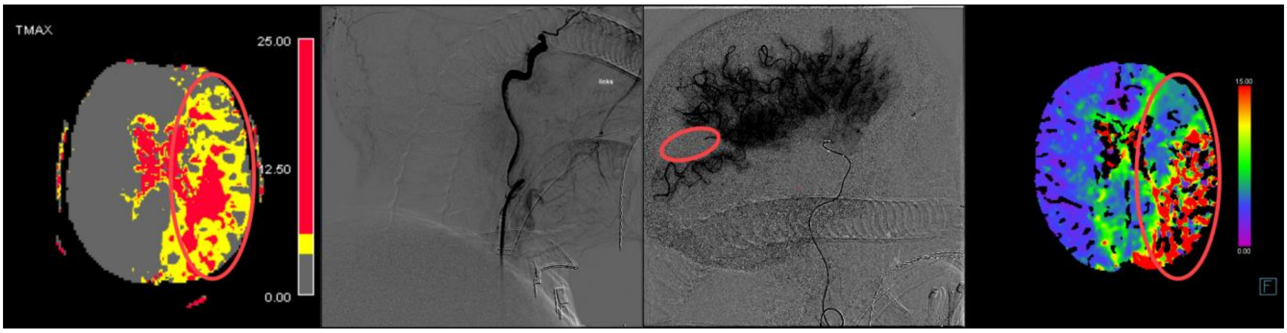

Figure 17. Far left: Admission perfusion imaging showing an occlusion pattern of the ICA. Middle left: First angiography imaging showing an ICA occlusion Middle right: Final angiography imaging showing small non-perfused area (eTICI 2c). Far right: Follow-up perfusion imaging showing same ICA occlusion pattern just as the admission imaging.

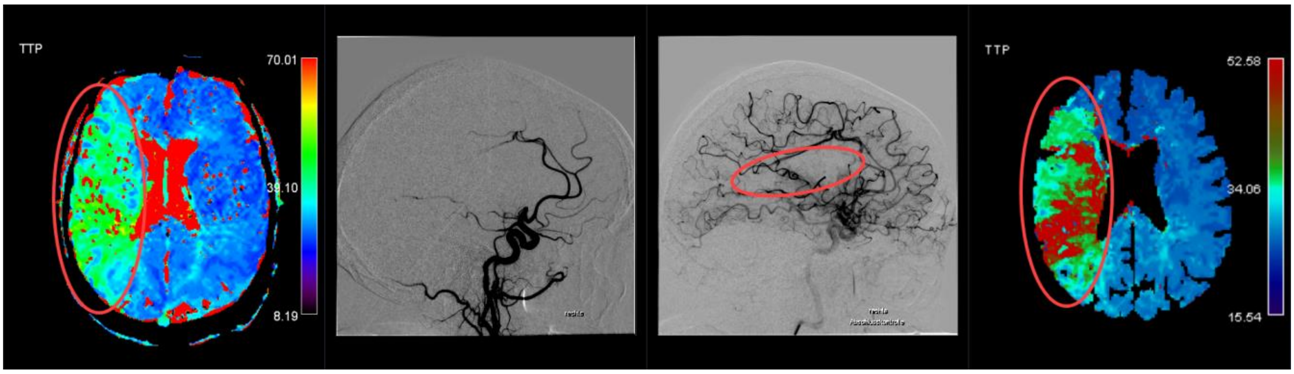

Figure 18. Far left: Admission perfusion imaging showing an occlusion pattern of the M1 occlusion. Middle left: First angiography imaging showing an M1 occlusion Middle right: Final angiography imaging partially reperfused area. Far right: Follow-up perfusion imaging showing same M1 occlusion pattern just as the admission imaging.

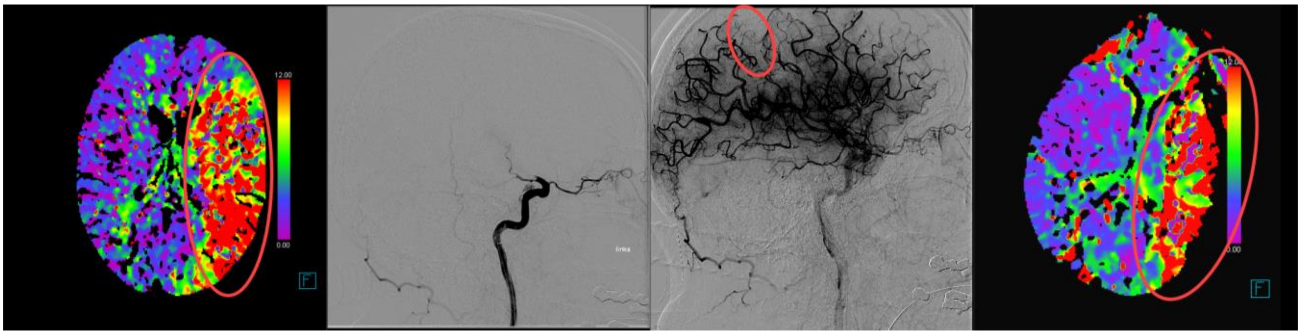

Figure 19. Far left: Admission perfusion imaging showing an occlusion pattern of an ICA occlusion. Middle left: First angiography imaging showing an ICA occlusion Middle right: Final angiography imaging partially almost complete reperfusion. Far right: Follow-up perfusion imaging showing same ICA occlusion pattern just as the admission imaging.

- **Emboli in new territory**

Thromboembolic migrations in a previously unaffected territory detectable on the final angiography run (defined as emboli to the new territory - ENT) also present themselves as perfusion delays. Hypoperfused areas which do not correspond to the target downstream territory of the primary vessel occlusion are not classified as true PPD.

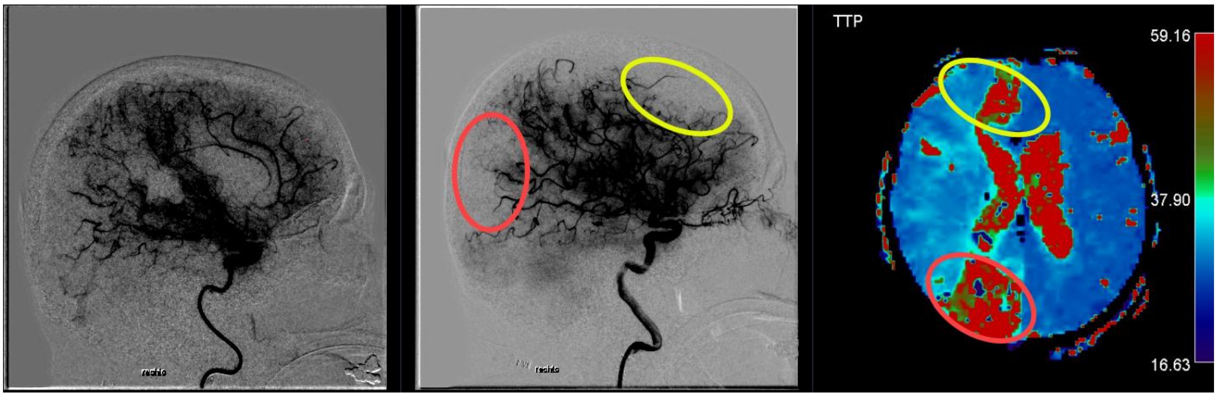

Figure 20. Left: Initial angiography imaging of an M1occlusion. Middle: Final angiography imaging showing residual non-perfused deficits. Right: Follow-up perfusion imaging showing PPD (red circle) which directly corresponds to the non-perfused angiography area, and emboli to new territory (yellow circle) corresponding to the newly occluded territory not observable on the initial angiography imaging.

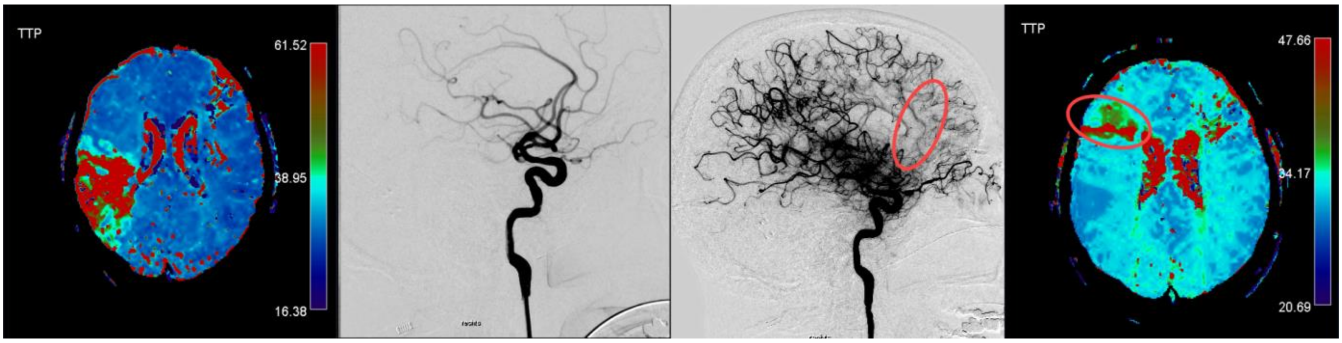

Figure 21. Far left: Initial perfusion imaging Middle left: Initial angiography imaging of an M2 occlusion. Middle right: Final angiography imaging showing completely reperfused MCA branch with new occlusion in the ACA territory (red circle). Far right: Follow-up perfusion

imaging showing emboli to new territory (red circle) directly corresponding to the ACA territory observable on the initial angiography imaging.

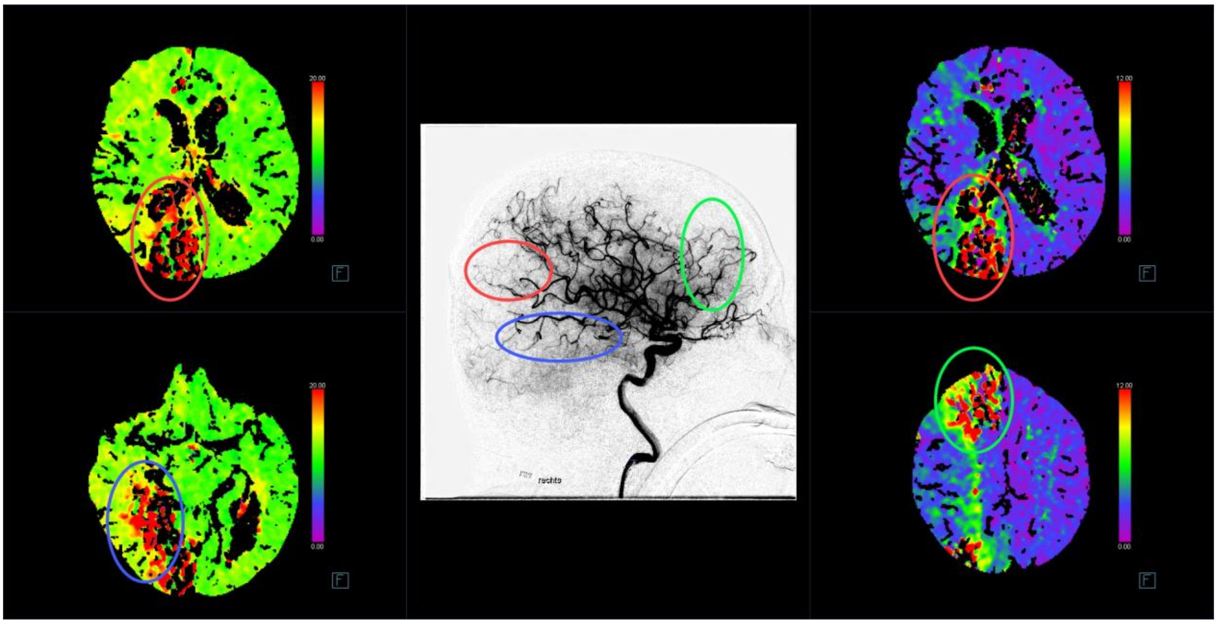

Figure 22. Middle: Patient initially presented with an M1 occlusion. Final angiography imaging showing incomplete reperfusion (eTICI 2b67) in the MCA territory (red circle), with emboli to new territories in the A3 (green circle) and P3 (blue circle) branches. Upper left: Follow-up TTP perfusion imaging showing persistent perfusion deficit due to distal M3 occlusion (red circle). Lower left: Follow-up TTP perfusion imaging showing emboli to new territory in the P3 territory (blue circle). Upper right: Follow-up Tmax perfusion imaging showing persistent perfusion deficit due to distal M3 occlusion (red circle). Lower right: Follow-up Tmax perfusion imaging showing emboli to new territory in the A3 territory (green circle).

- **Sustained reperfusion**

All patients who have achieved complete reperfusion at the final angiography run (eTICI 3) had sustained complete reperfusion on the 24-hour follow up imaging without any perfusion abnormalities.

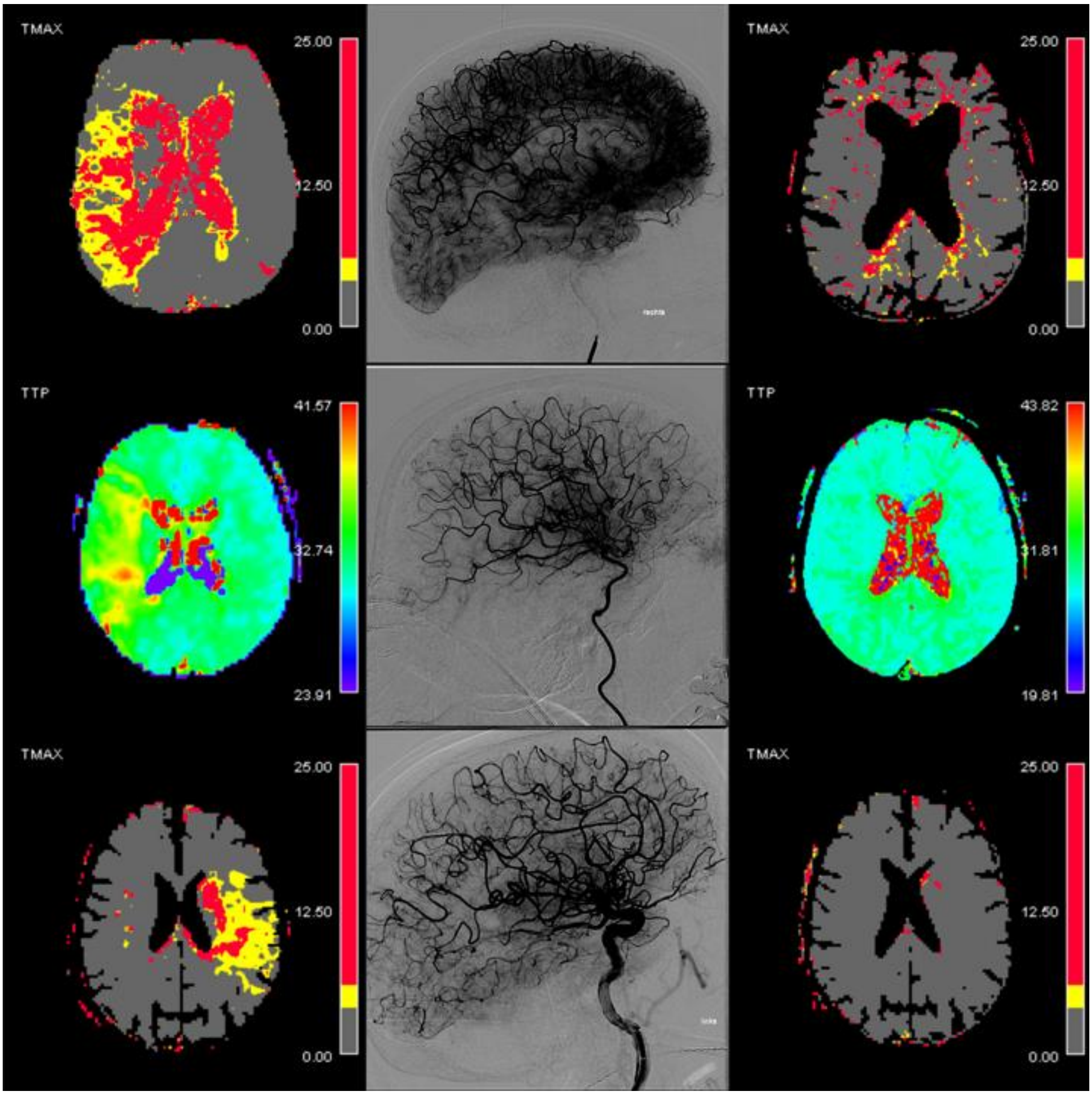

Figure 23: Left side: Perfusion imaging on admission. Middle: Final angiography run. Right side: Perfusion imaging on the 24 hour follow-up.

References

1. Wouters A, Christensen S, Straka M, Mlynash M, Liggins J, Bammer R, Thijs V, Lemmens R, Albers GW, Lansberg MG. A comparison of relative time to peak and tmax for mismatch-based patient selection. *Front. Neurol.* 2017;8:1–7.

2. Zaro-Weber O, Moeller-Hartmann W, Siegmund D, Kandziora A, Schuster A, Heiss WD, Sobesky J. MRI-based mismatch detection in acute ischemic stroke: Optimal PWI maps and thresholds validated with PET. *J. Cereb. Blood Flow Metab.* 2017;37:3176–3183.

3. Zaro-Weber O, Fleischer H, Reiblich L, Schuster A, Moeller-Hartmann W, Heiss WD. Penumbra detection in acute stroke with perfusion magnetic resonance imaging: Validation with 15O-positron emission tomography. *Ann. Neurol.* 2019;85:875–886.

4. Amukotuwa SA, Wu A, Zhou K, Page I, Brotchie P, Bammer R. Distal Medium Vessel Occlusions Can Be Accurately and Rapidly Detected Using Tmax Maps. *Stroke*. 2021;3308–3317.

5. Amukotuwa SA, Fischbein NJ, Albers GW, Davis S, Donnan GA, Andre JB, Bammer R. Comparison of T2*GRE and DSC-PWI for hemorrhage detection in acute ischemic stroke patients: Pooled analysis of the EPITHET, DEFUSE 2, and SENSE 3 stroke studies. *Int. J. Stroke*. 2020;15:216–225.
